# Inhibition of autophagy increases head and neck cancer sensitivity to cetuximab and radiation therapy

**DOI:** 10.1038/s41598-026-53573-6

**Published:** 2026-05-28

**Authors:** Yong-Syu Lee, Kwangok P. Nickel, Samantha T. Bradley, Justin H. Skiba, Jaimee C. Eckers, Adam D. Swick, Rong Hu, Mari Iida, Zafer Gurel, Deric L. Wheeler, Randall J. Kimple

**Affiliations:** 1 Department of Human Oncology, School of Medicine and Public Health, University of Wisconsin, University of Wisconsin, Madison, WI USA; 2 Department of Pathology and Laboratory Medicine, School of Medicine and Public Health, University of Wisconsin, University of Wisconsin, Madison, WI USA; 3 School of Medicine and Public Health, University of Wisconsin Carbone Cancer Center, University of Wisconsin, University of Wisconsin, Madison, WI USA

**Keywords:** Cancer, Cell biology, Oncology

## Abstract

The five-year survival rate of head and neck squamous cell carcinoma (HNSCC) remains around 50% despite advances in treatments over the last 20 years. Cetuximab, a monoclonal antibody targeting the epidermal growth factor receptor (EGFR), demonstrates limited efficacy both alone and in combination with chemotherapy, with response rates below 20% and 35%, respectively. This underscores the critical need for novel therapeutic strategies and a better understanding of treatment resistance mechanisms in HNSCC. Autophagy, a cell survival process essential to cellular stress for both normal and cancer cells, may contribute to therapeutic resistance in HNSCC. We hypothesized that current treatments might inadvertently activate autophagy, enabling tumor cells to evade cell death. We demonstrated that basal autophagy is elevated in cetuximab-resistant cells using an isogenic pair of cetuximab-sensitive and -resistant cell lines. Furthermore, combining cetuximab or radiation therapy with SAR405, an autophagy inhibitor, significantly enhanced growth inhibition both in vitro and in vivo compared to either treatment alone. Knockdown of LAPTM4B or EGFR significantly reduced cetuximab-induced autophagy. In contrast, radiation-induced autophagy is primarily driven by mitochondrial autophagy (mitophagy). Diminished radiation-induced autophagy was observed by the knockdown of PINK1, a key regulator of mitophagy. These findings highlight that inhibition of autophagy can improve the efficacy of HNSCC treatments and suggest that targeting specific autophagy subtypes may be crucial for developing personalized treatment combinations for HNSCC patients.

**Supplementary Information:**

The online version contains supplementary material available at 10.1038/s41598-026-53573-6.

## Introduction

Head and neck cell carcinoma (HNSCC) is the 6th most common cause of cancer death in the U.S. and is a major health issue worldwide^1^. Radiation therapy (RT) is a primary treatment option for many HNSCC patients. Cetuximab, monoclonal EGFR antibody, was first approved for treatment of HNSCC in combination with conventional treatments in 2006^2^ and continues to be used this day due to high expression of EGFR in the majority of HNSCC tumors^3,4^. Despite multimodality care, 5-year survival rates for patients with locally advanced HNSCC remain poor^5^. There remains significant room for improvements in treatment therapy. Concurrent chemoradiation is commonly recommended to improve both local and distant control^6^. The addition of cetuximab to standard RT enhances cure rates for newly diagnosed patients with HNSCC by 10%, however it is most often used only for those patients who are unable to tolerate cisplatin-based combinations. Cetuximab is also used in the metastatic setting where approximately 36% of patients respond to cetuximab-based combination therapy, compared to 20% who respond to standard therapy^7–12^. This indicates that nearly two-thirds of HNSCC patients do not respond to cetuximab-based therapy and highlights the need for a better understanding of therapeutic resistance and the development of novel treatment options for these patients.

Autophagy is an evolutionarily conserved cell survival mechanism utilized in response to multiple cellular stresses^13^. Autophagy begins when the Beclin-1/Vps34 complex generates phosphatidylinositol 3-phosphate (PI3P) pools to nucleate the phagophore. During vesicle elongation, LC3B-I is lipidated to form LC3B-II, which integrates into the autophagosomal membrane to facilitate cargo sequestration. The efficiency of this process is measured as autophagic flux, the rate at which these vesicles fuse with lysosomes for substrate degradation. EGFR signaling has been involved in autophagy. Tan et al. demonstrated that inactive EGFR forms a complex with LAPTM4B, trapping Rubicon and releasing Beclin1, thereby leading to autophagy induction^14,15^. This suggests that cetuximab-induced EGFR inactivation may inadvertently promote cell survival by activating autophagy. While RT has also been shown to promote autophagy, its precise role in HNSCC remains unclear^16^. There is a long history of investigating autophagy inhibitors as anti-cancer drugs. Chloroquine and hydroxychloroquine, which prevent lysosomal acidification and thus block autophagy in vitro, have been investigated as anti-cancer agents in over 50 clinical trials (clinicaltrials.gov) due to their autophagy inhibiting properties^17–19^. However, initial results from some studies have been underwhelming. Autophagy plays a complex role in cancer, both promoting cancer development and protecting against cellular stress induced by anti-cancer therapies. This dichotomy emphasizes the need to determine the role of autophagy in HNSCC and to understand how to effectively combine autophagy modulators with existing treatments.

Herein, we present clear evidence highlighting the role of autophagy in HNSCC cells and tumors treated with cetuximab or RT and its contribution to therapeutic resistance. In HNSCC patients, tissue microarray (TMA) analysis demonstrated that LC3 expression is elevated in more advanced stage primary cancers. Combination therapy utilizing SAR405, as an autophagy inhibitor, enhanced the response to both cetuximab and RT in vitro and in a cell line xenograft model of HNSCC. Finally, we identified the specific subtype of autophagy activated by cetuximab and RT to understand the mechanism of autophagy induction by these treatments.

## Results

### Cetuximab and radiation increase autophagy in head and neck cancer

**Fig. 1:**
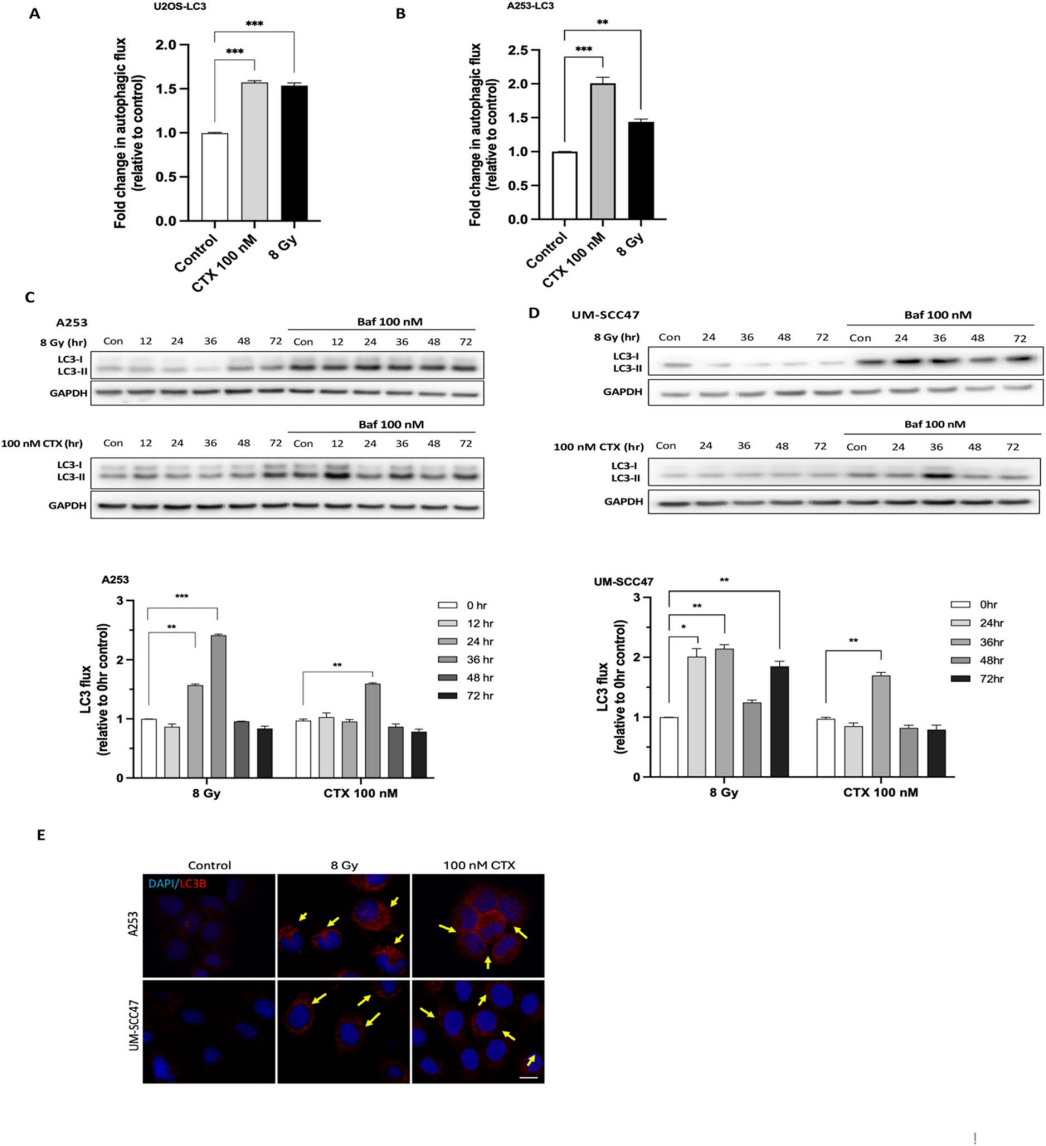
Cetuximab (CTX) and irradiation (RT) increase autophagy. **(A)** The U2OS-LC3 cell line showed an increase in autophagic flux 36 h after treatment with either 8 Gy of radiation or 100 nM CTX, as measured by the Autophagy LC3 HiBiT Reporter Assay (Promega). **(B)** A stable salivary gland carcinoma cell line, A253-LC3, was generated by transfecting A253 cells with the LC3 HiBiT reporter vector. Treatment with either 8 Gy or 100 nM CTX significantly increased autophagic flux by 36 h post-treatment in the A253-LC3 cells, a result similar to that observed in U2OS-LC3 cells. (C) A253 and (D) UM-SCC47 cells were treated with 100 nM CTX or 8 Gy to determine the time point that maximally induces autophagy. To evaluate LC3 levels, a western blot was performed. LC3 flux was subsequently measured by calculating the ratio of LC3-II expression with and without Bafilomycin A1 (Baf) treatment, after normalizing to GAPDH and then to the control. Unprocessed scans of blots are available in supplemental information. (E) Immunofluorescent (IF) staining was performed to visualize autophagy. Both HNSCC cell lines, A253 and UM-SCC47, exhibited LC3 puncta formation after 36 h treatment with 8 Gy or 100 nM CTX. To promote accumulation of LC3 puncta, the cells were treated with 100 nM Baf for 4 h before fixation. DAPI and LC3B were imaged in separate channels and reassembled with pseudo coloring. LC3 puncta are indicated by a yellow arrow in the images. Scale bar, 20 μm. Columns, mean; bars, standard error of the mean (*n* = 3). *, *P* < 0.05, **, *P* < 0.01, ***, *P* < 0.001, according to t test (control to treatment, or 0 h to other time points).

To determine whether autophagy is modulated by common treatments for HNSCC, we first examined the autophagy levels in cetuximab (CTX) and radiation (RT) treated cells. We used an LC3-based reporter assay (Autophagy LC3 HiBiT Reporter Assay, Promega), to investigate autophagic flux in U2OS LC3-reporter cells (U2OS HiBiT LC3, Promega). Both CTX (100 nM) and RT (8 Gy) increased autophagic flux following treatment, indicating they induced autophagy (Fig. 1A). To confirm these findings in HNSCC cells, we generated a stable A253-LC3 reporter cell line. Both CTX and RT again elevated autophagic flux compared to control treatment (Fig. 1B). We next measured LC3 flux in un-transfected A253 cells using immunoblotting in cells treated with either CTX or RT. Bafilomycin A1 (Baf) was used to assess LC3 flux by comparing the level of LC3-II. Both CTX and RT induced LC3flux (Fig. 1C). Similar findings were also found in the HNSCC cell line UM-SCC47 (Fig. 1D). Assessment of LC3B puncta using immunofluorescence staining following either CTX or RT treatment again verified the induction of autophagy (Fig. 1E). Altogether, these findings demonstrate both CTX and RT induce autophagy in HNSCC cell lines.

### Autophagy leads to cell survival in HNSCC

**Fig. 2:**
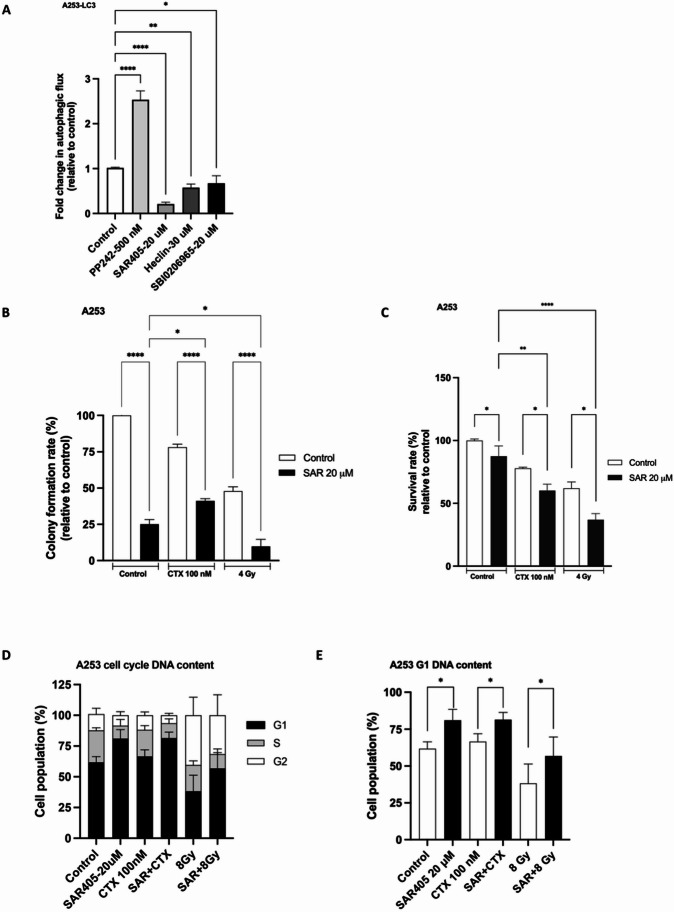
Autophagy serves as a cell protector in HNSCC. (**A**) A253-LC3 reporter cells were treated for 48 h with the indicated doses of putative autophagy inhibitors and inducer. The Autophagy LC3 HiBiT Reporter Assay (Promega) was then used to measure their autophagy effect. (**B**) A253 cells were pre-treated with 20 µM SAR405 for 6 h prior to treatment with 100 nM CTX or 4 Gy. Medium was refreshed every 3 days until the 12th day. The colony forming rate was measured as the ratio of counted colonies to seeded cells and normalized to the control, which suggested an inhibitory effect of SAR405 in colony formation. (**C**) Proliferation of A253 cells was assessed using CCK-8 assay 72 h after indicated treatment. (**D&E**) A253 cells were pre-treated with 20 µM SAR405 6 h prior to the co-treatment of 100 nM CTX or 4 Gy. DNA content was measured by flow cytometry at 36 h post-treatment. The flow cytometry results indicate that SAR405 induces cell cycle arrest at the G1phase (**E**). Columns, mean; bars, standard error of the mean (*n* = 3). *, *P* < 0.05, **, *P* < 0.01, ***, *P* < 0.001, ****, *P* < 0.001, according to t test (control to treatment).

Autophagy has been linked to both cell survival and cell death^20^. We examined autophagic flux of small-molecule inhibitors targeting distinct stages of the autophagy pathway using the A253-LC3 reporter cell line(Fig. 2A). PP242, an ATP-competitive mTOR inhibitor, induces robust autophagy by providing a complete blockade of both mTORC1 and mTORC2, including the rapamycin-resistant 4E-BP1 target. SAR405, a selective Vps34 inhibitor, prevents PtdIns3P production and subsequent autophagosome membrane expansion. Heclin stabilizes autophagy-related proteins by inhibiting HECT E3 ubiquitin ligases (i.e. NEDD4), thereby preventing the proteasomal degradation of the Vps34/Beclin-1 complex. Finally, SBI-0206965, a selective ULK1 inhibitor, prevents the phosphorylation of ATG13 and Beclin-1. Treatment with PP242 enhanced autophagic flux by 2.5 fold, while SAR405, Heclin, and SBI-0206965 each inhibited the flux (0.21, 0.57, and 0.67 fold, respectively). SAR405 demonstrated the strongest inhibitory effect. From this result, the role of SAR405 was further examined. To determine the specific role of autophagy in HNSCC following CTX or RT treatment, we evaluated cell survival using two different methods: a clonogenic survival assay and a tetrazolium-based cell proliferation assay. SAR405 decreased colony formation (Fig. 2B) but had relatively modest effects on proliferation (Fig. 2C). The addition of SAR405 to RT treatment resulted in a further reduction in colony formation when compared to either RT or SAR405 alone suggesting that inhibition of autophagy with SAR405 lowered HNSCC cell survival (Fig. 2B). Colony formation with the combined CTX and SAR405 was greater than that with SAR405 alone (Fig. 2B), suggesting possible divergent mechanisms by which CTX and RT activate autophagy.

When SAR405 was combined with RT, cell survival was further reduced compared to CTX combination in both cell proliferation and clonogenic survival assays. To better understand the effect of SAR405 on cell proliferation, we investigated how treatment combinations affected cell cycle distribution using flow cytometry (Fig. S1). This result indicated that SAR405 arrested cells in the G1 phase regardless of the treatment (Fig. 2D and E). These data suggest that autophagy appears to play a protective role in cell survival.

### Enhanced autophagy in Cetuximab resistant HNSCC cells and malignant tissues

**Fig. 3:**
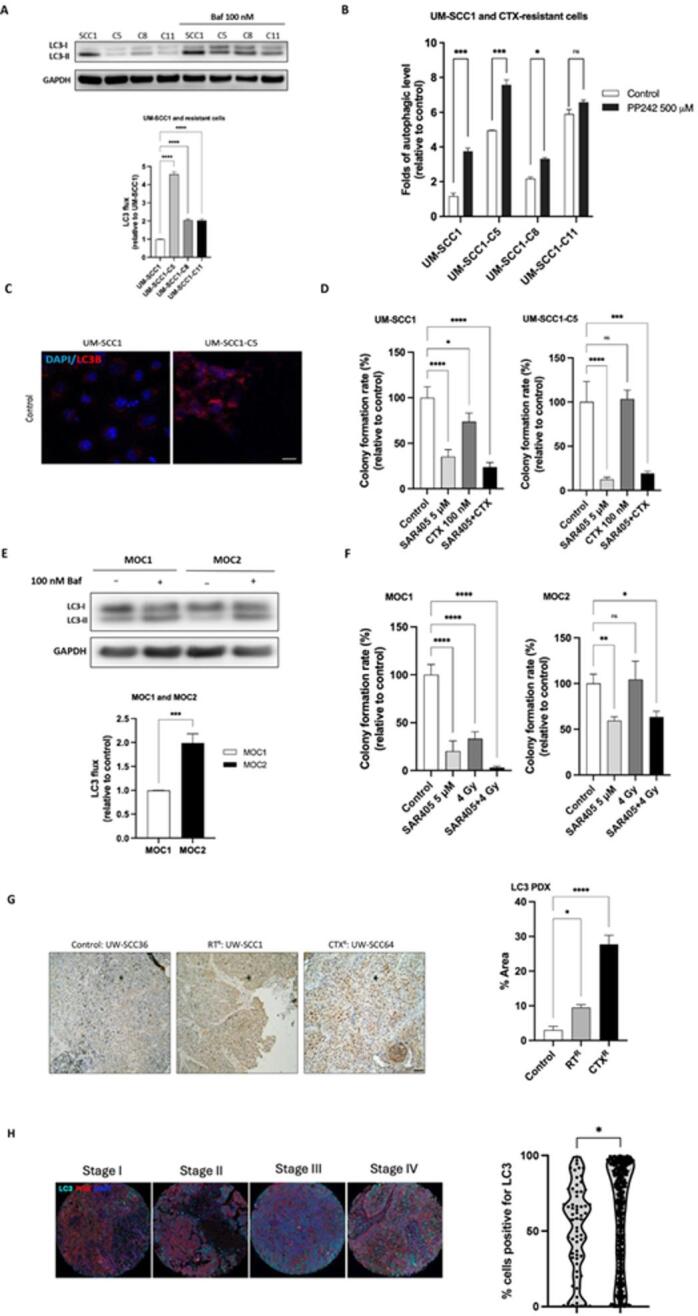
Cetuximab-resistant cell lines have higher basal autophagy levels. **(A**) LC3 flux was measured in UM-SCC1 and its cetuximab (CTX)-resistant derivative cell lines (UM-SCC1-C5/C8/C11) by immunoblotting. Bafilomycin A1 (Baf) was added 4 h before protein extraction to induce LC3-II accumulation for the flux calculation. The flux as shown in the lower panel demonstrates increased autophagy in the CTX-resistant cells. Unprocessed scans of blots are available in supplemental information. (B) Acridine orange was used to quantify the numbers of autophagosome, and the data was normalized to cell counts. Cells treated with PP242 for 24 h showed increased autophagy levels, indicating these resistant cells can respond to the drug. **(C)** IF staining of UM-SCC1 and UM-SCC1-C5 cells treated with 8 Gy or 100 nM CTX for 36 h exhibited LC3 puncta formation. Cells were treated with 100 nM Baf 4 h prior to fixation to accumulate LC3 puncta. Scale bar, 20 μm. **(D)** Cetuximab-sensitive (UM-SCC1) and resistant (UM-SCC1-C5) cells were treated with 100 nM CTX, 5 µM SAR405, or control for 7 days and then clonogenic survival was assessed. **(E)** Radiation resistant cell line (MOC2) was assessed its basal autophagy flux compared to the sensitive cell line (MOC1) using immunoblotting analysis. Unprocessed scans of blots are available in supplemental information. **(F)** Radiation-sensitive (MOC1) and RT-resistant (MOC2) cells were treated with 4 Gy, 5 µM SAR405, or control for 7 days and then stained with crystal violets for colonies. Colony numbers were all normalized to untreated control. **(G)** Tumor sections from different patient-derived xenografts (PDX) were stained with LC3B using immunohistochemistry method. More LC3B positive cells have been observed in both CTX-resistant and RT-resistant tissues. Scale bar, 100 μm. **(H)** The analysis of TMA over 173 patients were used to determine the LC3 status in different malignant stage patient tissues. Samples were stained with LC3 and pan-cytokeratin (PCK) and imaged by Vectra imaging system. The algorithm was established to calculate the LC3B area detected only in tumor tissues (LC3 and PCK double positive) but not in stromal tissues. Columns, mean; bars, standard error of the mean (*n* = 3). *, *P* < 0.05, **, *P* < 0.01, ***, *P* < 0.001, ****, *P* < 0.001, according to t test (UM-SCC1 to its resistant clones, control to treatment, and MOC1 vs. MOC2).

Once we identified the role of autophagy as a cellular protector, we investigated its effects in HNSCC cells and patient-derived xenograft tissues that exhibit resistance to standard therapies. We utilized a panel of cells with acquired resistance to CTX originally established by the Wheeler lab^21^. These cells were treated with increasing concentrations of CTX to establish resistant clones. Immunoblotting analysis showed greater LC3 flux in CTX resistant cell lines (UM-SCC1-C5, -C8, and -C11) when compared to their parental cell line UM-SCC1 (Fig. 3A). We also demonstrated that these resistant cell lines were able to further activate autophagy by treating them with the mTOR inhibitor, PP242 which acts as an autophagy activator (Fig. 3B). Immunofluorescence staining also exhibited increased LC3B puncta in CTX resistant cells UM-SCC1-C5 than in parental UM-SCC1 cells (Fig. 3C). Colony formation was assessed to confirm resistance to CTX in the resistant cell UM-SCC1-C5 (Fig. 3D). Inhibition of autophagy with SAR405 significantly decreased colony formation in both CTX sensitive and CTX resistant cells (Fig. 3D). MOC1 and MOC2 are isogenic oral cancer cell lines derived from a murine host^22–24^. Since they do not express human EGFR, both are not responsive to CTX. MOC2 is known to be more radioresistant than MOC1^25^. Like the CTX resistant cell line results, increased LC3B flux was observed in radiation resistant MOC2 cells when compared to the radiation sensitive MOC1 cells (Fig. 3E). SAR405 sensitized the radiation-sensitive MOC1 cells to radiation. However, MOC2 cells showed no radiation response, but exhibited decreased survival when treated with SAR405, independent of RT (Fig. 3F). Immunohistochemistry staining of LC3B in patient-derived xenograft (PDX) tissues to resistant to CTX or RT^26–28^ was evaluated. Patient tissues with CTX or RT resistance exhibited a higher LC3B expression when compared to the patient tissues that were sensitive to these treatments (Fig. 3G). These findings suggest that autophagy may protect HNSCC from CTX and RT leading to therapeutic resistance.

There is no straightforward way to assess LC3 flux in fixed patient tissues as LC3 IHC or multiplex immunofluorescence (IF) staining captures only a single moment in time. Despite this limitation, a TMA consisting of samples from 173 HNC patients were evaluated by IF multiplex staining for LC3 to determine whether we could correlate LC3 expression with stage at diagnosis (Fig. 3H). Using a cytokeratin (PCK) mask to assess only tumor tissue, we quantified the percentage of LC3 expression only in cancer cells. LC3 expression was higher in primary tumors from patients with advanced Stage III or IV cancer than in those with early Stage I or II cancer (18.79% (*n* = 21) vs. 8.16% (*n* = 152)). This TMA data suggest an important role for autophagy in tumor progression.

### Combining SAR405 with CTX/RT augments the anti-cancer effect in vivo

**Fig. 4:**
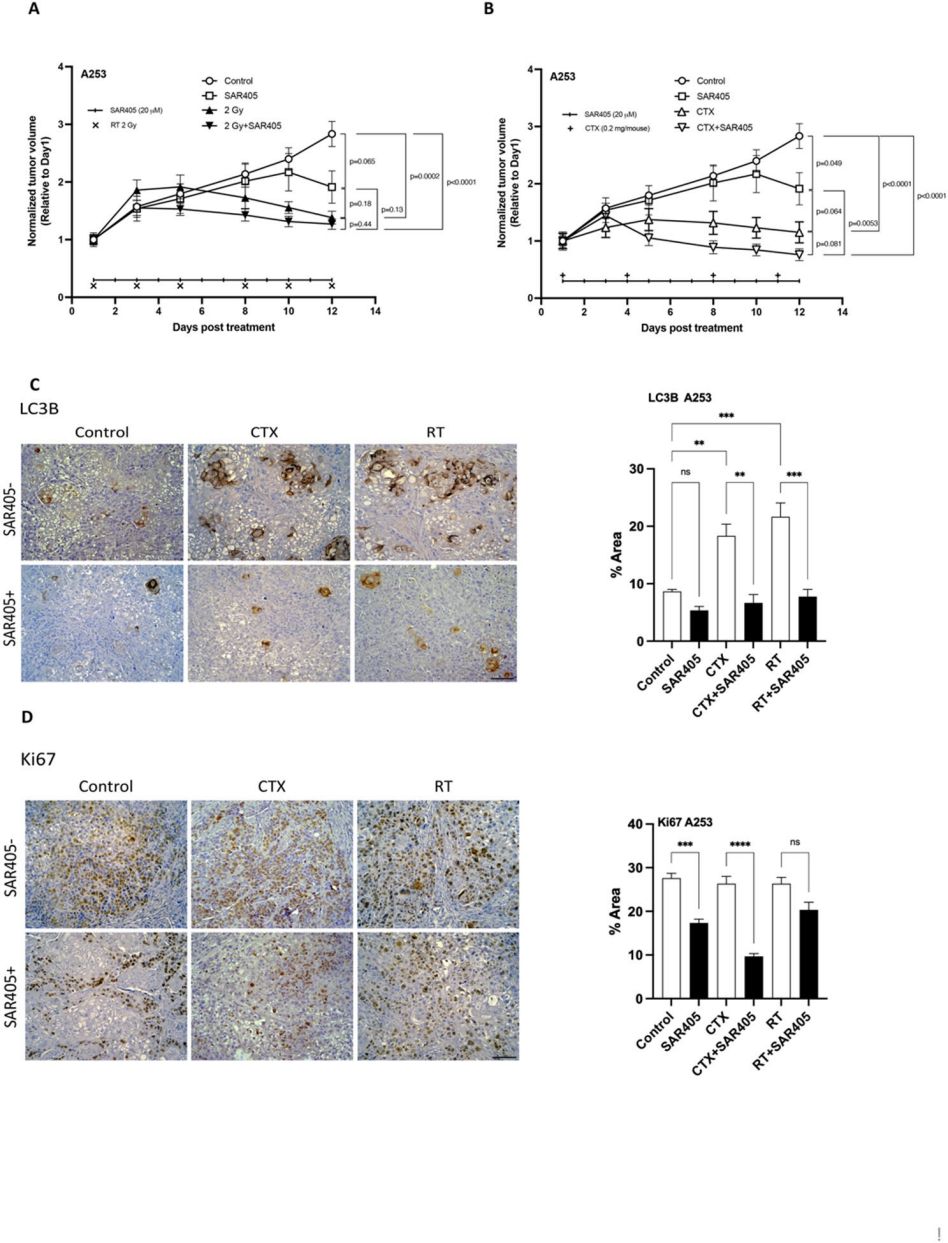
Combining SAR405 with CTX/RT augments the anti-cancer effect in vivo. **(A-B)** A253 tumors were pre-treated with 20 µM SAR405 6 h prior to the treatment of CTX (0.2 mg/mouse, 2 days/wk) or RT administration (3 Gy, 3 days/wk). Mice were treated with SAR405 by intra-tumoral injection every day. Tumor sizes were measured every other day with caliper. Tumor tissues were collected 72 h post-implantation for IHC evaluation. **(C)** IHC of LC3B demonstrated more LC3B positive cells in both CTX- and RT-treated mice, however, SAR405 significantly decreased numbers of LC3B positive cells. Scale bar, 100 μm. **(D)** IHC staining for Ki67, a proliferation marker, was used to confirm that SAR405 decreased cell proliferation through cell cycle arrest. Scale bar, 100 μm. Columns, mean; bars, standard error of the mean (*n* = 3). Points, mean tumor volume in mice after treatment; bars, standard error of the mean (*n* = 10–12 tumors/group). *, *P* < 0.05, **, *P* < 0.01, ***, *P* < 0.001, according to t test (control to treatment).

We established A253 cell xenografts to investigate whether autophagy inhibition improves tumor control when combined with RT or CTX in vivo. Then tumors were treated with the autophagy inhibitor SAR405 in combination with control, CTX, or RT. While SAR405 had little effect alone (Fig. 4A and B), its combination with RT resulted in slightly enhanced tumor growth inhibition compared to RT alone although there is no significant statistical difference was observed (Fig. 4A). Similarly, the combination of CTX and SAR405 led to a trend towards improved tumor regression compared to CTX treatment alone (*p* = 0.081, Fig. 4B). Tumors were harvested 72 h after treatment stained for LC3B and Ki67, a cell proliferation marker. IHC staining for LC3B showed increased expression of LC3B in CTX and RT treated animals and confirmed that SAR405 inhibits autophagy in vivo (Fig. 4C). SAR405 decreased cell proliferation as measured by Ki67 expression (Fig. 4D) in CTX treated animals, but did not significantly decrease Ki67 expression in combination with RT. Taken together, these findings suggest the use of SAR405, or other autophagy inhibitors could have a role in the treatment of HNC to improve the efficacy of CTX or RT.

### EGFR/LAPTM4B mechanism governs CTX-induced autophagy

**Fig. 5:**
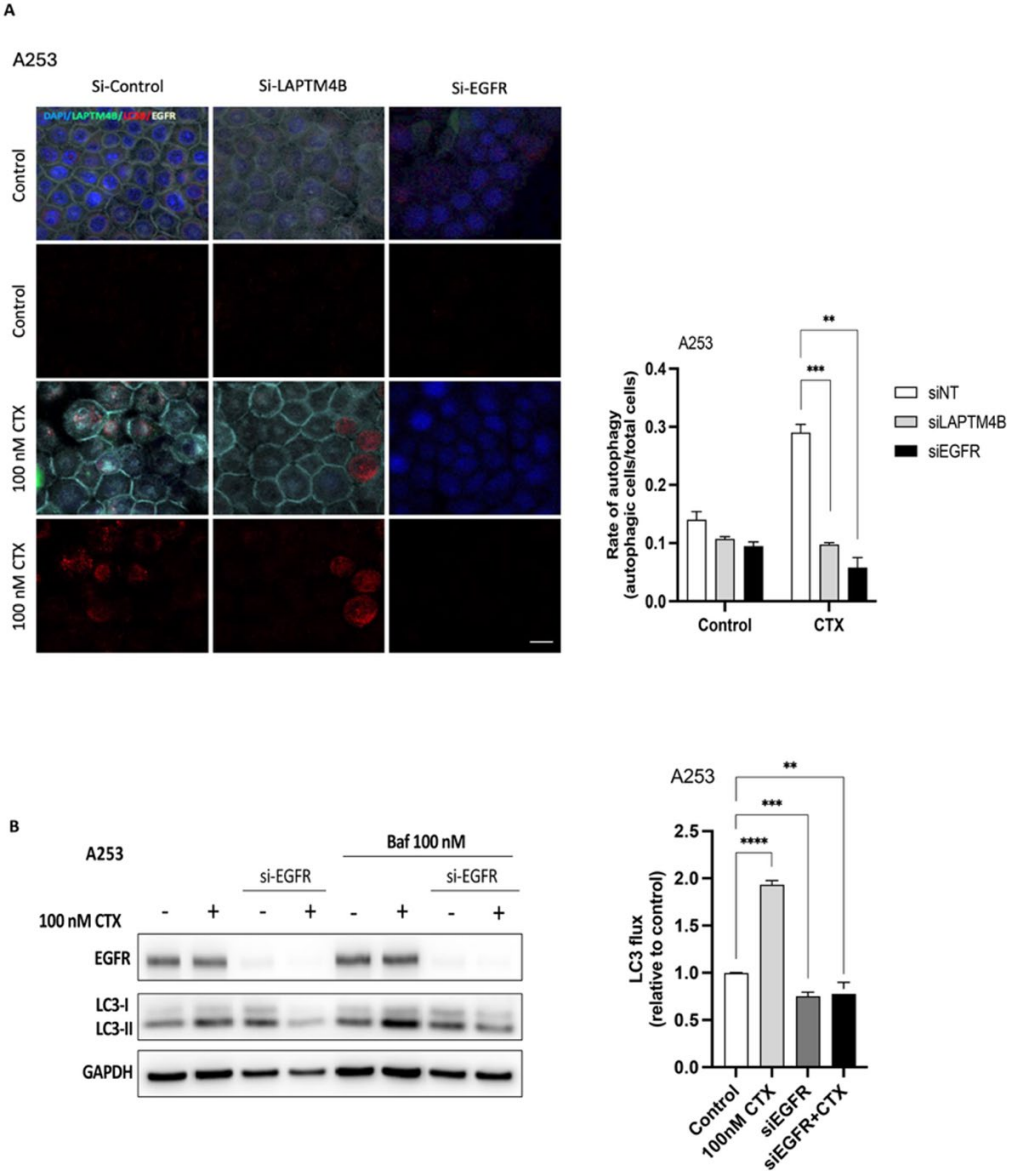
EGFR and LAPTM4B are involved in CTX-induced autophagy. **(A)** Immunofluorescence staining of LAPTM4B and LC3 revealed that both proteins co-localized to EGFR containing endosomes in A253 cells treated with 100 nM CTX for 36 h. 4 h prior to fixation, cells were treated with 100 nM Baf to accumulate LC3 puncta. Knockdown of either EGFR (25 nM) or LAPTM4B (50 nM) was sufficient to disrupt this interaction. Numbers of autophagic cells have been normalized to cell counts. A253 cells were pre-treated with siRNAs for 12 h and then treated with 100 nM CTX another 36 h. Scale bar, 20 μm. **(B)** Immunoblotting analysis for LC3B in the presence or absence of si-EGFR was used to confirm the effect of EGFR knockdown on LC3 flux. Unprocessed scans of blots are available in supplemental information. Columns, mean; bars, standard error of the mean (*n* = 3). *, *P* < 0.05, **, *P* < 0.01, ***, *P* < 0.001, according to t test (control to treatment).

Inactive EGFR has been shown to form a complex with LAPTM4B to prevent Rubicon from interacting with the autophagy initiator Beclin1, thus leading to autophagy initiation^14,15^. Therefore, we hypothesized that CTX-induced autophagy is dependent on the EGFR/LAPTM4B interaction due to the role of CTX as an EGFR inhibitor. To test this, we first confirmed that transfection with knockdown of EGFR or si-LAPTM4B effectively silenced their respective protein and mRNA targets in HNSCC cells (Fig. S2). Knockdown of EGFR or LAPTM4B significantly decreased autophagy as assessed by LC3B puncta and LC3 flux (Fig. 5A and B), suggesting that LAPTM4B is involved in CTX-induced autophagy. We next investigated the protein-protein interaction between Rubicon and Beclin1 to determine whether CTX induced autophagy is mediated by the EGFR/LAPTM4B machinery. Our findings showed that levels of Beclin1 co-immunoprecipitated with Rubicon were significantly lower in CTX-treated cells compared to the control (Fig. S3). This suggests that the EGFR/LAPTM4B complex promotes autophagy by sequestering Rubicon, thereby releasing more Beclin. Collectively, these results suggest that CTX activates autophagy in a LAPTM4B, Rubicon, and Beclin1 dependent manner.

### RT-induced autophagy is mitophagy controlled by PINK1/PARKIN signaling pathway

**Fig. 6:**
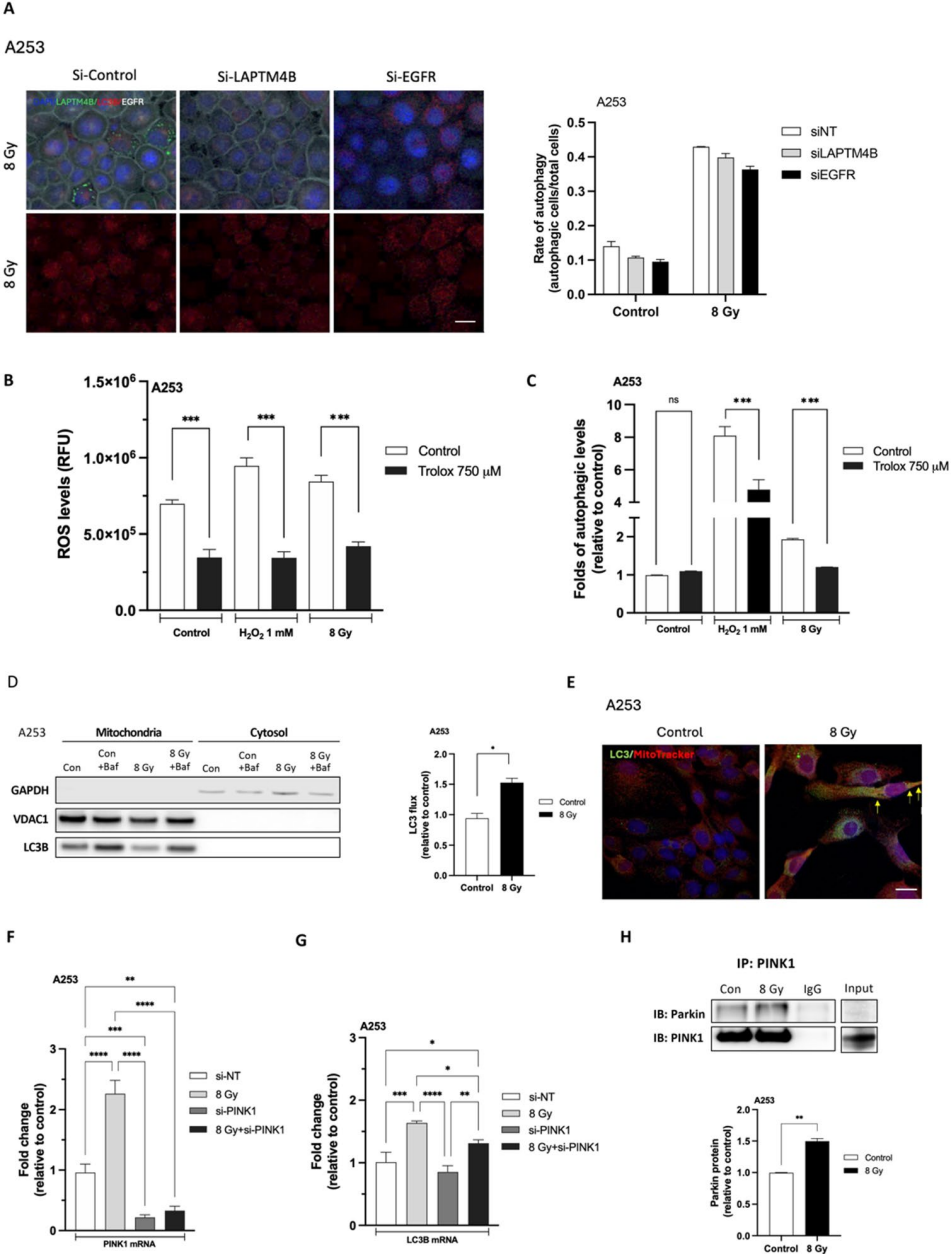
RT-induced autophagy is mTOR signaling dependent but not involved in EGFR and LAPTM4B. **(A)** Immunofluorescence staining of LC3 revealed that there is no significant difference of autophagy by 8 Gy 36 h post treatment in A253 cells. 4 h prior to fixation, cells were treated with 100 nM Baf to accumulate LC3 puncta. Scale bar, 20 μm. **(B)** Reactive oxygen species (ROS) levels have been increased by 36 h post treatment with H_2_O_2_ at 1 mM and 8 Gy treatment in A253 cells compared to control, and ROS levels were all decreased by pretreating cells with superoxide radical scavenger, Trolox for 6 h. ROS levels were measured using CM-H2DCFDA ROS dye. **(C)** Radiation-induced ROS can activate autophagy as measured with acridine orange. **(D)** Higher autophagy was found in mitochondria (mitophagy) at 36 h following 8 Gy in A253 cells compared to control using immunoblotting. 4 h prior to protein extraction, Baf were added to accumulate LC3 to calculate the LC3 flux. Unprocessed scans of blots are available in supplemental information. **(E)** Immunofluorescence staining of LC3 (green) combined with mitoTracker RED CMXRos (red) revealed significant colocalization of signal at 36 h post 8 Gy treatment in A253 cells. 4 h prior to fixation, cells were treated with 100 nM Baf to accumulate LC3 puncta. Scale bar, 20 μm. **(F)** Pink1 mRNAs were increased by 36 h post 8 Gy treatment in A253 cells. Knockdown of Pink1 using siPink1 at 25 nM significantly inhibited Pink1 mRNA expressions even in irradiated A253 cells. **(G)** LC3 mRNA was upregulated by 8 Gy but downregulated by siPINK1, indicating that RT-induced autophagy is involved in PINK1 signaling pathway. **(H)** Co-immunoprecipitation assay was performed to understand the protein-protein interaction between Pink1 and Parkin. Cells were irradiated with 8 Gy for 36 h followed by fresh cell lysis using NP40 lysis buffer. Anti-rabbit IgG was used as the negative control. Input was run separately as clearly delineated. Unprocessed scans of blots are available in supplemental information. Columns, mean; bars, standard error of the mean (*n* = 3). *, *P* < 0.05, **, *P* < 0.01, ***, *P* < 0.001, ****, *P* < 0.001, according to t test (control to treatment).

Since CTX-induced autophagy was related to EGFR/LAPTM4B machinery, and RT has been shown to result in alteration of EGFR signaling, we were intrigued to examine whether RT-induced autophagy shared the same mechanism. To test this hypothesis, we again transcriptionally inhibited LAPTM4B and EGFR using si-LAPTM4B or si-EGFR. Our findings showed no effect on autophagy between control and the cells with EGFR/LAPTM4B knockdown (Fig. 6A), suggesting that RT-induced autophagy is modulated by alternative pathways.

RT induces significant reactive oxygen species (ROS) which leads to mitochondrial stress^29^, an effect that could activate autophagy. Autophagy levels were examined using acridine orange dye combined with the ROS scavenger, Trolox. Irradiated cells demonstrated an increase in ROS that can be blocked by treatment with Trolox (Fig. 6B). Trolox treatment blocks autophagy induction by RT or ROS generated by H_2_O_2_ treatment (Fig. 6C) strongly suggesting that RT-induced autophagy is mediated by ROS generation. We assessed mitochondrial and cytoplasmic LC3B expression in cellular fractionation extracts and showed that RT-induced autophagy was concentrated in the mitochondria (Fig. 6D). LC3B flux in response to radiation was mostly detected in mitochondria using immunoblotting (Fig. 6D). These results were confirmed by immunofluorescence for LC3B (green) and MitoTracker (red) (Fig. 6E).

Recent evidence suggests that PINK1 (PTEN-induced putative kinase 1) and Parkin signaling regulates the induction of mitophagy^30,31^. We quantified PINK1 mRNA levels by qRT-PCR and found that RT (Fig. 6F), but not CTX (Fig. S4), significantly upregulated PINK1 expression. Knockdown of PINK1 successfully abrogated this RT induced increase of PINK1 (Fig. 6F). Furthermore, PINK1 silencing suppressed the RT mediated elevation of LC3B mRNA (Fig. 6G). To investigate the underlying mechanism, we performed co-immunoprecipitation assays, which revealed an enhanced association between PINK1 and Parkin following irradiation. More Parkin was pulled down with PINK1 following radiation, suggesting that radiation activates the PINK1/Parkin interaction (Fig. 6H). Altogether, these data indicate that PINK1 cooperates with Parkin to facilitate RT induced mitophagy in HNSCC.

## Discussion

Autophagy not only controls cellular homeostasis but also plays a critical role in cancer development leading from healthy cells to metastatic or drug-resistant tumors. This complex regulation highlights the opportunities for pharmacologic intervention in the multi-step process of autophagy. However, the precise role of autophagy in cancer remains highly complex. Autophagy has been shown to play a role in both evasion of cell death and maintenance of homeostasis through cellular recycling programs, and in the promotion of cell death through large-scale degradation of cellular components^32,33^. This dual role of autophagy poses an immense challenge to anti-cancer treatment approaches and suggests the understanding of the mechanism by which individual treatments modulate autophagy is critical. Drug-induced autophagy has been employed as an attempt to kill cancer cells, particularly in those that have adopted anti-apoptotic strategies^34–36^. Meanwhile, an opposite approach has been taken wherein autophagy is inhibited as an attempt to overcome the treatment resistance conferred by autophagy^37,38^. It should be noted that there are currently no FDA approved compounds that were developed specifically to inhibit autophagy. Rather, autophagy modulation was discovered as an off-target effect of the drugs used in these studies. We are therefore actively investigating whether chemotherapy and radiotherapy can be enhanced with the addition of autophagy modulators to improve tumor control and prevent the development of therapeutic resistance, as autophagy has numerous effects on cancer treatments.

In this work, we demonstrate that autophagy is activated in HNSCC cells treated with CTX or RT. In HNSCC, autophagy plays a protective role to cell death caused by these common treatments. We observed therapy induced autophagy in both HPV-negative and HPV-positive HNSCC cells but determining the precise role for HPV oncoproteins in this process is beyond the scope of this manuscript. In both acquired and intrinsic resistance models, autophagy was higher than in related sensitive lines. This is consistent with the finding of increased autophagy in patients with more advanced stages of HNSCC. We acknowledge the limitation that LC3B immunohistochemistry in fixed tissues only provides a snapshot and does not permit one to comment on autophagic flux in these tissues. This highlights the unmet need of a clinically straightforward biomarker for autophagy measurement from patients.

Chloroquine (CQ) and Hydroxychloroquine (HCQ) are currently the only two autophagy inhibition compounds be tested in clinical trials. Although there were several convincing experimental data in support of their anti-cancer effects^39^, the results of clinical trials have shown limited efficacy^40^. These compounds act at the late stages of autophagy by interfering with autolysosome formation, which may account for their limited clinical efficacy^41^. The late stage blockade allows for the accumulation of stalled autophagic vacuoles and often fails to fully halt metabolic recycling. Therefore, the clinical translation of autophagy inhibition warrants the development of more potent stage specific inhibitors. In this study, we evaluated the anticancer potential of SAR405, a specific inhibitor that targets autophagy at the initiation stage. Unlike late-stage inhibitors, SAR405 targets the Vps34-dependent Class III PI3K complex, which is essential for the nucleation of the pre-autophagosomal phagophore and precludes the formation of autophagosomes entirely. This early-stage intervention prevents cells from sequestering cytoplasmic components for nutrient recovery, thereby inducing a profound metabolic crisis. Our findings demonstrate that as a monotherapy, SAR405 suppresses proliferation by inducing cell cycle arrest at the G1 phase without exerting cytotoxic effects. Moreover, when combined with either CTX or RT, SAR405 enhanced growth inhibition compared to monotherapies both in vitro and in vivo. SAR405 also improved the control of therapy resistant cells suggesting that early-stage autophagy inhibition sensitizes cells to cellular stress. By targeting alternative autophagic processes may provide a synergistic boost to inhibitory effects. Two such candidates currently under investigation are the ULK1 inhibitor SBI-0206965 and the NEDD4-1 inhibitor Heclin, though their capacity to sensitize tumors to CTX and RT remains to be fully elucidated.

**Fig. 7:**
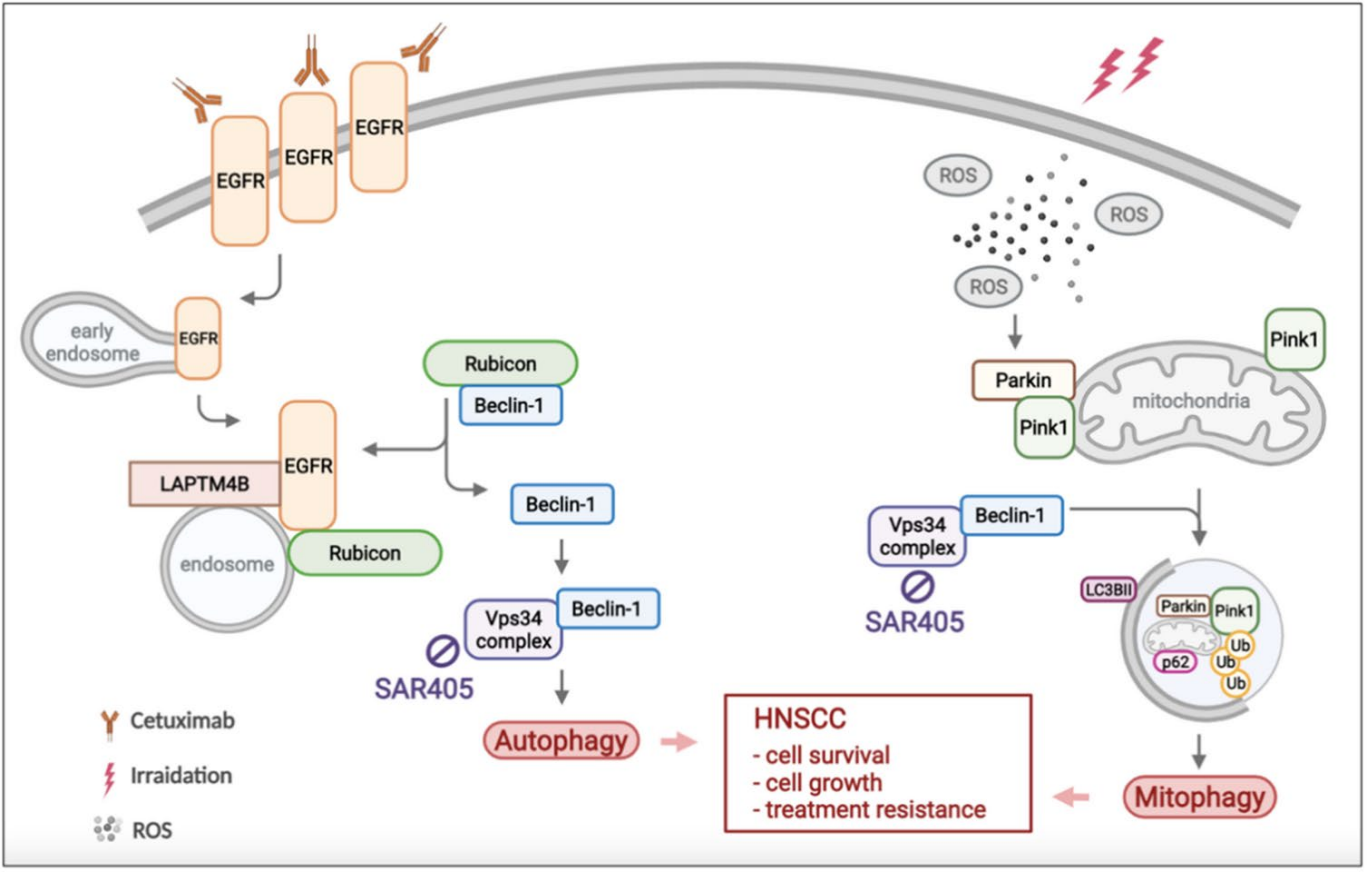
Graphic summary of CTX and RT-induced autophagy.

Previous studies have shown that various cellular stresses activate autophagy via the interaction between inactive EGFR and LAPTM4B^14^. Given that CTX inhibits and downregulates EGFR, we explored how this mechanism relates to CTX induced autophagy. Whether this process occurs in other CTX sensitive cancers remains a subject worthy of further study. Interestingly, radiation induced autophagy was found to function independently of this EGFR/LAPTM4B machinery. Instead, PINK1/Parkin mediated mitophagy activation was linked to the radiation induced autophagy in HNSCC. These findings emphasized that there are various types of autophagy that occur in HSNCC in response to different cellular stresses and cancer treatments (Fig. 7).

In conclusion, our work suggests that CTX and RT both induce autophagy in HNSCC. Furthermore, CTX and RT resistant cells demonstrate increased autophagy compared to sensitive cells, suggesting autophagy plays a role in the development of therapeutic resistance. We have demonstrated that CTX and RT activate autophagy through independent mechanisms but that inhibition of autophagy using a VPS34 inhibitor can improve tumor control in vivo.

## Materials and methods

### Cell lines and drug

Human head and neck cancer cell lines (HPV-positive: UM-SCC47; squamaous cell carcinoma of the oral tongue, HPV-negative: A253; salivary gland carcinoma, UM-SCC1; squamous cell carcinoma of the oral cavity) were obtained from the UW Head and Neck SPORE cell line repository. Cetuximab resistant cell lines, UM-SCC1-C5, -C8, and -C11 derived from UM-SCC1, were kindly obtained from Dr. Deric Wheeler. Syngeneic C57BL/6 (B6) mouse OSCC cell lines, MOC1 and MOC2, were kindly provided by Dr. Ravi Uppaluri (Dana-Farber Cancer Institute). All cells were cultured at low passage in standard conditions (at 37 °C in 5% CO2). All human HNSCC cells were cultured in Dulbecco’s modified Eagle’s Medium (DMEM) (Corning Inc.) supplemented with 10% fetal bovine serum, 1% penicillin/streptomycin, 4 mM L-glutamine, 4.5 g/L glucose, and 1 mM sodium pyruvate. Mouse cell lines (MOC1 and MOC2) were maintained in modified DMEM/F12 at a 2:1 mixture with 5% fetal bovine serum, 1% penicillin/streptomycin, 5 ng/mL epidermal growth factor, 400 ng/mL hydrocortisone, and 5 µg/mL insulin. Cetuximab (Erbitux) purchased from UW Heath Pharmacy, was stored at 4 °C. SAR405 was purchased from APExBIO (Houston, TX). SBI-0206965 (#SML 1540) and Heclin (SML 1396) were from Sigma-Aldrich. PP242 (#S2218) was from Selleckchem (Houston, TX).

### Immunoblot analysis

Cells were harvested and washed with PBS before freezing at -80 °C. Then, cells were lysed with RIPA buffer supplemented 1% (v/v) with phosphatase/protease inhibitor cocktail (Cell Signaling Technologies, #5872) and sonicated. Equal amounts of protein were analyzed by SDS-PAGE, transferred to polyvinylidene difluoride membranes, and probed with specific primary antibodies overnight at 4 °C. Protein targets of interest were detected with NIR-conjugated anti-mouse and anti-rabbit secondary antibodies (LiCOR) and imaged on a LiCOR Odyssey FC. Specific antibodies and sources are listed in Table S1. For autophagy flux analysis, after certain drug treatments, cells were treated with or without 100 nM bafilomycin A1 (Baf) 4 h prior to cell lysis. Densitometric analysis of bands was performed using Image Studio software (LiCOR). The signal intensities of the LC3-II and GAPDH bands were quantified, and then the LC3-II signal was normalized to the corresponding GAPDH loading control. Autophagic flux was calculated as the change in normalized LC3-II levels between Baf-treated and untreated condition. Many blots were cut prior to hybridization as was the standard in our lab at the time the experiments were performed. Unprocessed scans of blots are available in supplemental information and include the full extent of available membranes. Due to an upgrade of our gel documentation system after completion of the studies, but before submission of the manuscript to this journal additional images and/or exposures are no longer available.

### Irradiation

Cells were irradiated with an Xstrahl X-ray System, Model RS225 (Xstrahl, UK) at a dose rate of 3.27 Gy/minute at 30 cm FSD, tube voltage of 195 kV, current of 10 mA and filtration with 3 mm Al. Animals were irradiated with an X-RAD320 (Precision X-Ray, North Branford, CT) with 1 Gy/minute delivered at 320 kV/12.5 mA at 50 cm FSD with a beam hardening filter with half-value layer of 4 mm Cu. The delivered dose rate was confirmed by ionization chamber. Mice were shielded with custom-built lead jigs to limit radiation exposure to the rear quarter of the body.

### LC3B puncta immunofluorescence (IF) staining

Cells were plated in 8-well chamber slides at densities ranging from 10,000 to 30,000 cells/well depending on cell type size and growth rate. Twenty-four hours post-plating cells were treated with control, 100 nM CTX, or irradiated with 8 Gy and incubated at 37 °C in 5% CO2 for 36 h. 100 nM bafilomycin A1 (Baf) was added to cells 4 h prior to fixing cells with 100% methanol in PBS at indicated time points. Cells were permeabilized with 0.1% Triton-X 100 in TBST for 15 min, blocked with goat serum for 1 h at 25 °C, and incubated with anti-LC3B primary antibody overnight at 4 °C (Table S1). Cells were then probed with Alexa Fluor 555 conjugated secondary antibody (CST #4413) for 1 h at 25 °C in the dark, and cover-slipped with Fluoromount containing DAPI. The next day, they were imaged at 60x magnification using a Nikon A1RS inverted point scanning confocal microscope system.

### Imaging cytometry-based autophagy flux assay

Cells were seeded in black-wall, clear-bottom 96-well plates. After overnight incubation, cells were treated with and without 100 nM Baf for 24 h. For the image-cytometer based acridine orange (AO) assay, 24 h post-treatment, the plates were washed three times (100µL/well) with 1X PBS. 50µL of 1 µg/ml Acridine Orange (AO) was added to each well and the plates were incubated at room temperature for 30 min. Cells were then washed two times with 1X PBS. 50µL of 1X PBS was added per well, and then the plates were read on the SpectraMax i3x Multi-Mode Microplate Reader Platform with MiniMax 300 Imaging Cytometer (Molecular Devices, Sunnyvale, CA) using the SoftMax Pro software (v6.4). The monochromator and MiniMax settings were used on the software. The monochromator setting was used to measure excitation/emission wavelengths of 500/526 (green) to assess intensity of unprotonated, diffuse AO staining DNA (non-autophagic staining), and 460/650 (red) to assess intensity of dimerized, protonated AO concentrated in acidic vesicular organelles (autophagic staining)^42^. The MiniMax setting was used to count the number of cells per well as we have previously described^43^. The red intensity signal per well was divided by the green intensity or the cell count to assess the level of autophagy and to assess the efficacy of the two normalization methods: controlling to green intensity as recommended by Thome et al. vs. controlling to cell count as previously done by Fowler et al.^42,43^.

### Autophagy LC3 HiBiT reporter assay

Autophagic flux was assessed using the autophagy LC3 HiBiT reporter system (Promega). A253 cells were transfected with LC3 HiBiT reporter vector (#GA255A) using Lipofectime 2000 (Themo Fisher Scientific), and the stable A253-LC3 cell line was generated by G418 selection. U2O2 cells (#GA1050), and A253 cells expressing the HiBiT-tagged LC3 protein were seeded in 96-well plates and treated with 100 nM CTX or 8 Gy irradiation at indicated times. Following treatment, LC3 degradation was quantified by the addition of a Nano-Glo HiBiT lytic reagent containing the LgBiT protein and the Nano-Glo HiBiT lytic substrate. The complementation of HiBiT and LgBiT resulted in a bioluminescent signal proportional to the amount of remaining LC3 protein. Luminescence was measured using SpectraMax i3x Microplate Reader (Molecular Devices). Fold change in autophagic flux was calculated as bioluminescent signal normalized to the untreated control. To more clearly demonstrate the autophagic flux changes we took the reciprocal of the resulting fold change in figures (Fig. 1A and B, and 2A).

### Clonogenic survival assay

Cells were seeded into 6-well plates at specific densities, incubated overnight, and treated with indicated treatment (100 nM CTX, 4 Gy, or control). Once colonies averaged 50 or more cells (7–10 days) in the control wells, plates were fixed and stained with 1% (w/v) crystal violet in methanol, and colonies of 50 or more cells were counted. Survival bar graphs were generated after normalizing to the amount of cell death in control group using Prism.

### Proliferation assay

Cells were plated in 96-well plates at densities ranging from 1,000 to 3,000 cells/well according to cell type growth rate. Twenty-four hours post-plating, cells were treated with 100 nM CTX, 20 µM SAR405, 4 Gy irradiation, or DMSO control and incubated for 3 days. Once control wells neared full confluence, Cell Counting Kit-8 (CCK8) reagent was added (Dojindo Molecular Technologies) and absorbance measured at 450 nm on a SpectraMax i3x microplate reader (Molecular Devices). The absorbance of treated wells was normalized to control wells.

### Cell cycle analysis

Cells were plated in 6 cm dishes at densities ranging from 200,000 to 600,000 cells/well depending on the cell type and growth rate. Twenty-four hours post-plating, cells were treated with 100 nM CTX, 8 Gy irradiation, 20 µM SAR405 or DMSO control, incubated for 36 h, and trypsinized and cells were collected. Cells were centrifuged at 500 x g for 10 min, washed with PBS, pelleted again, and a single cell suspension was created in 0.5 ml PBS. 4.5 ml chilled 70% methanol was added to each tube and cells were stored in 4 °C. Twenty-four hours later, cells were centrifuged at 500 x g for 10 min, and a single cells suspension was created in 1 ml solution of propidium iodide (Molecular probes # P3566), Triton X-100 (0.1%) (Sigma #T9284), and RNase A (Thermo Scientific #EN0531) in PBS. The samples were analyzed using the Attune NxT flow cytometer (Thermo Scientific), and the cell cycle distribution was calculated using ModFIT software (Verity Software House, Top-sham, ME).

### Cell line xenograft growth delay study

Six to eight-week old female Hsd: athymic Nude-Foxn1nu (Harlan Laboratories) mice were used for growth delay studies. Mice were kept in the Association for Assessment and Accreditation of Laboratory Animal Care-approved Wisconsin Institute for Medical Research Animal Care Facility. Studies were carried out in accordance with an animal protocol approved by the University of Wisconsin-Madison Institutional Animal Care and Use Committee. All methods were carried out in accordance with relevant guidelines and regulations. A253 cells were grown in vitro, mixed 1:1 with Matrigel, and injected subcutaneously into bilateral flanks of nude mice at 1 × 10^6^ cells/site. Tumor volumes were measured twice weekly with digital vernier calipers and calculated using V = (π/6) x (large diameter) x (small diameter)^2^. Once average tumor size reached ~ 200 mm^3^, mice were randomized into treatment groups. Vehicle (1% (v/v) DMSO in saline) or CTX (0.2 mg) was administered twice per week by intraperitoneal injection. SAR405 (20 µM) was administered once daily by intratumoral injection. Radiation was delivered three times per week, 2 Gy per fraction. The treatment arm lasted for 12 days. Five to 7 mice (10–14 tumors) were used per treatment group with animals excluded if no tumors were established. At the end of the experiment, mice were euthanized according to approved protocol using 100% CO_2_ introduced at the rate of 30–70% of the chamber volume per minute. Death was confirmed by verifying cardiac and respiratory arrest. All methods are reported in accordance with ARRIVE guidelines.

### Immunohistochemistry (IHC)

Five µm sections from formalin fixed paraffin embedded samples were deparaffinized with xylene and hydrated through graded solutions of ethanol. Antigen retrieval was conducted in sodium citrate retrieval buffer (pH 6.0) followed by washing in running water. Slides were washed in PBS and then incubated with 0.3% hydrogen peroxide solution. Blocking was carried out using 10% goat serum in PBS and then incubated with the primary antibody of Ki67 and LC3B (Table S1) diluted in 1% goat serum in PBS containing 0.1% Triton X-100 overnight at 4 °C. Slides were washed with PBS next day; secondary antibody was used (SignalStain^®^ Boost IHC Detection Reagent HRP Rabbit, CST #8114). Staining was detected using diaminobenzidine (Vector Laboratories, Inc. #SK-4100). The slides were counterstained with 1:10 hematoxylin (Thermo Scientific #TA-125-MH) solution for 2 min, then dehydrated in ethanol and xylene solutions and sections were covered with coverslip with Cytoseal (Thermo Scientific #8312–4). Three sections were taken from three independent tumors for each control and experimental condition. Quantification of IHC staining was completed using Aperio ImageScope (12.3.3, Leica Biosystem) to identify areas of positive staining. Images were annotated to remove blank space, and the minimum intensity threshold was set using the control group for each experiment.

### Tissue microarray (TMA) LC3B immunostaining

Multiplex immunostaining for LC3B expression was assessed in 173 patients from the UW Head and Neck SPORE oropharynx tissue microarray treated between 1989 and 2017 using the Opal method following the manufacturer’s protocol (Akoya, CA), and the antibodies were applied in sequence as follows: LC3A/B/Opal 620 (Abcam, ab128025, dilution factor of 1:3000) and pan-cytokeratin (PCK)/Opal 690 (Abcam, ab7735, dilution factor of 1:2000). Stained tissue microarray slides were then scanned with Vectra 2 (Akoya, CA) at 20X. InForm Tissue Analysis Workflow v.2.4. software (Akoya, CA) was used to create an automated algorithm to segment stroma and epithelium as well as identify LC3 expression. PCK staining was used to identify tumors and then exclude stromal tissues. Percentages for LC3 and PCK double positive area was determined.

#### Quantitative real time-polymerase chain reaction (qRT-PCR)

Total RNA was extracted from cultured cells using RNeasy Mini Kit (Qiagen, Valencia, CA) and measured by Nanodrop. RNA was used to synthesize cDNA with SuperScrip III First-Strand Synthesis System (Invitrogen, Carlsbad, CA). qRT-PCR was performed using PowerUP SYBR Green master mix (Applied Biosystems) with primers listed in Supplemental Table 2. Relative mRNA levels were quantified (Bio-Rad, S1000 Thermal cycler) and normalized to housekeeping gene GAPDH. All reactions were performed in triplicate from RNA isolated from three independent biological experiments.

### Co-immunoprecipitation (co-IP)

Cells were pelleted and lysed in NP40 buffer containing protease and phosphatase inhibitor cocktails immediately after the end of drug treatment. Cell lysates were kept on ice through the process. Syringes with 23G needles were used to physically breakdown cell membranes. Cells were spun down at 14,000 rpm at 4 °C for 30 min and supernatant was collected. 250ug-500 ug of proteins were used for the immune-precipitation using protein A/G plus agarose (Santa Cruz, #sc-2003). Indicated primary antibodies were used for immunoblotting analysis.

### Statistical analysis

A two-tailed Student’s t test was used to evaluate the differences between two groups. One-way ANOVA, followed by Dunnett’s or Holm-Sidak’s multiple comparisons test, was used for multiple group comparisons. Data were analyzed with Prism 8 (GraphPad Software). *P* < 0.05 was considered statistically significant. All experiments were repeated at least three times and presented as means ± SEM.

## Electronic Supplementary Material

Below is the link to the electronic supplementary material.

Supplementary Material 1

## Data Availability

All data are available in the main text or the supplementary materials.
