# Supplementary material for: Inhibition of autophagy increases head and neck cancer sensitivity to cetuximab and radiation therapy
Source: Sci Rep. 2026 May 28;16:24472. doi: 10.1038/s41598-026-53573-6 (PMC13451215; doi:10.1038/s41598-026-53573-6)

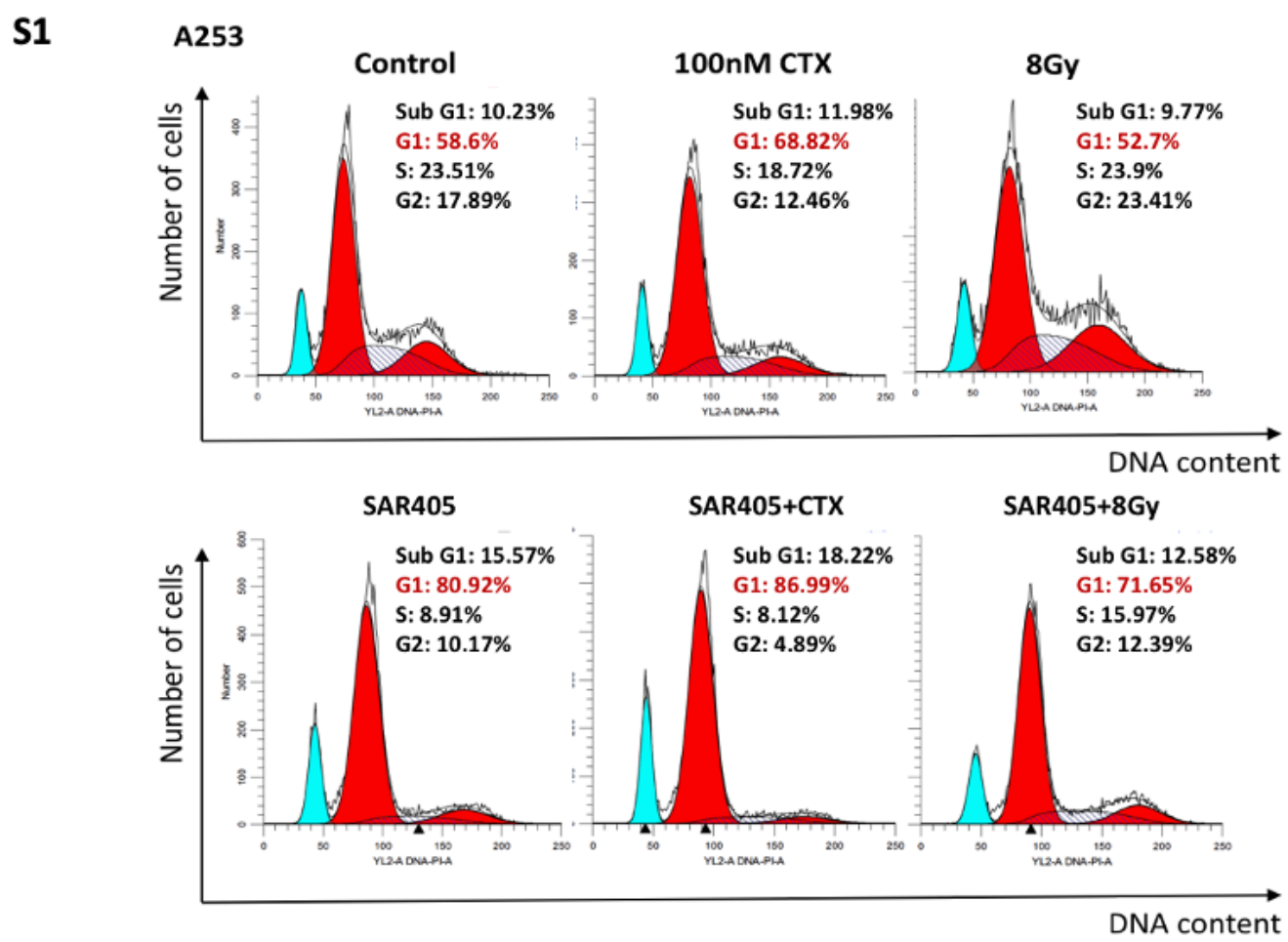

Fig. S1 SAR405 arrests cell cycle at G1 phase in HNSCC cells. Cells were treated with 100 nM CTX, 20 µM SAR405, 8 Gy, or DMSO control for 36 hr and then collected for the cell cycle analysis using flow cytometry. The population quantified results were based on channel gating and compared to untreated control.

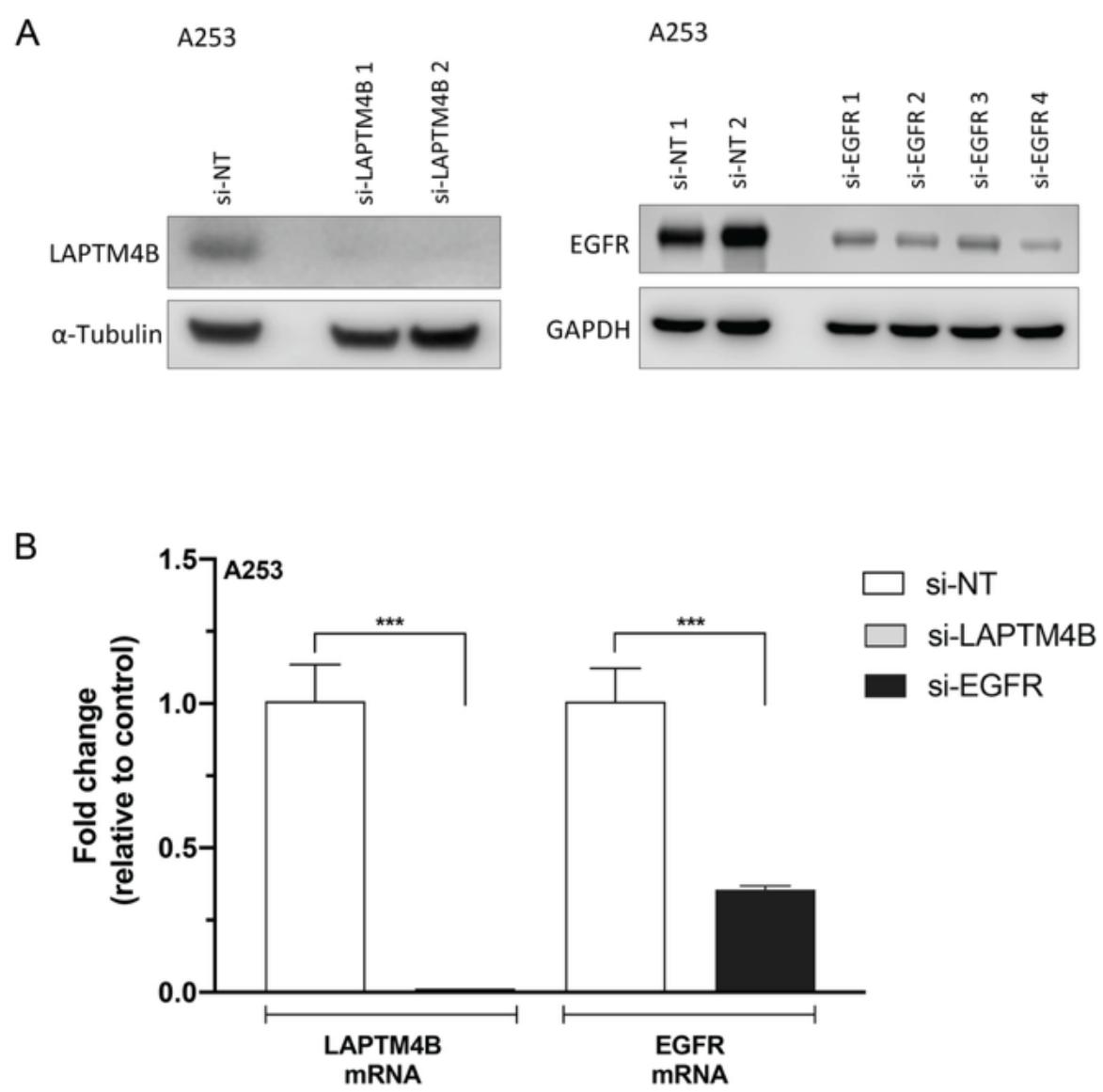

Fig. S2 Knockdown of LAPTM4B and EGFR. Western blot and qPCR was used to determine the efficiencies of knockdown by si-LAPTMB (50 nM) and si-EGFR (25 nM).

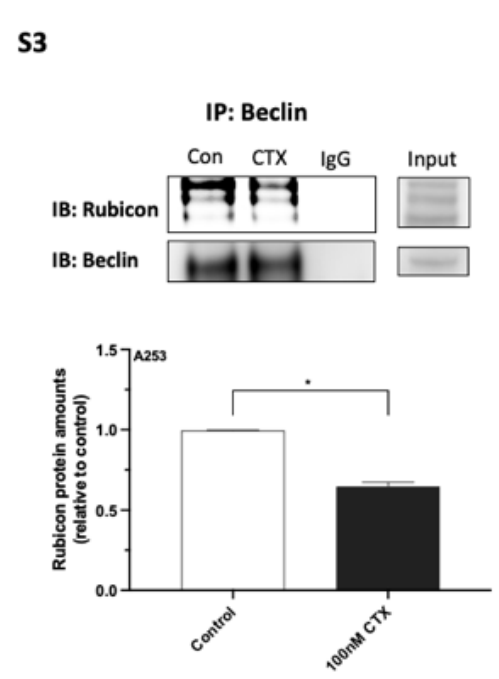

Fig. S3 Co-immunoprecipitation of Rubicon and Beclin1 following CTX. Immunoprecipitation was performed to understand the protein-protein interaction between Rubicon and Beclin1. Cells were treated with 100 nM CTX for 36 hr followed by fresh cell lysis using NP40 lysis buffer. Anti-rabbit IgG was used as the negative control and the lysate input was sed for the recognition of the bands.

S4

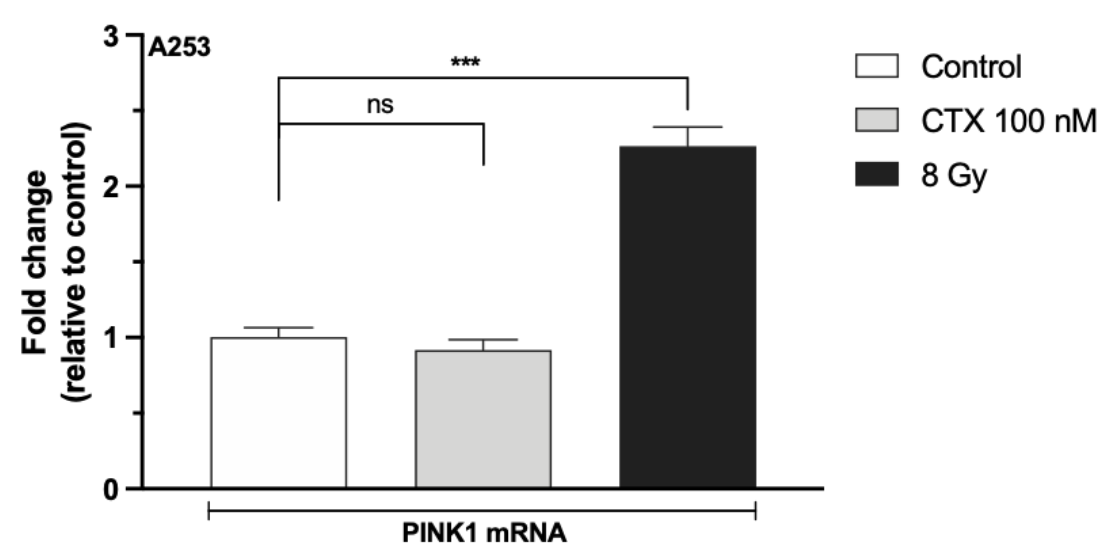

Fig. S4 Radiation but not CTX increases PINK1 mRNA. A253 cells were treated with 100 nM CTX or 8 Gy and Pink1 mRNA quantified by qRT-PCR 26 h after treatment.

Supplemental Table 1

Table 1. Antibody List					
**Antibodies**	**Concentration**	**Purpose**	**Host**	**Brand**	**Catalog number**
LC3B	1:1000	WB, IF (cell)	Rabbit	Cell Signaling	3868
LC3B	1:500	IHC	Rabbit	Cell Signaling	3868
Pink1	1:1000	WB	Rabbit	Cell Signaling	6946
Parkin	1:50	coIP	Mouse	Cell Signaling	4211
GAPDH	1:2000	WB	Rabbit	Cell Signaling	5274
LAPTM4B	1:1000-1:500	WB, IF (cell)	Rabbit	NOVUS	NBP2-61158
α-Tubulin	1:2000	WB	Mouse	Sigma Aldrich	CP06
EGFR	1:1000	WB, IF (cell)	Rabbit	Cell Signaling	4267
Ki67	1:400	WB, IF (cell)	Rabbit	Cell Signaling	9027
Rubicon	1:1000	WB	Rabbit	Cell Signaling	7151
Rubicon	1:50	coIP	Rabbit	Cell Signaling	7151
Beclin-1	1:1000	WB	Mouse	Cell Signaling	4122

Supplemental Table 2

Table 2 Primer List		
**h-LAPTM4B**	F	5'-GTT ACC AGC AAT GAC ACT ACGG-3'
	R	5'-CTC CTT GGC AGC ACC ATT-3'
**h-LC3B**	F	5′-GAG AAG CAG CTT CCT GTT CTG G-3′
	R	5′-GTG TCC GTT CAC CAA CAG GAA G-3′
**h-PINK1**	F	5′-GTG GAA CAT CTC GGC AGG TT-3′
	R	5′-CCT CTC TTG GAT TTT CTG TAA GTG AC-3′
**h-EGFR**	F	5'-GTG CAG CTT CAG GAC CAC AA-3'
	R	5'-AAA TGC ATG TGT CGA ATA TCT TGA G-3'
**18S**	F	5'-GTA ACC CGT TGA ACC CCA TT-3'
	R	5'-CCA TCC AAT CGG TAG TAG CG-3'

Unprocessed scanned blots matching to the figures

Fig. 1C

A253

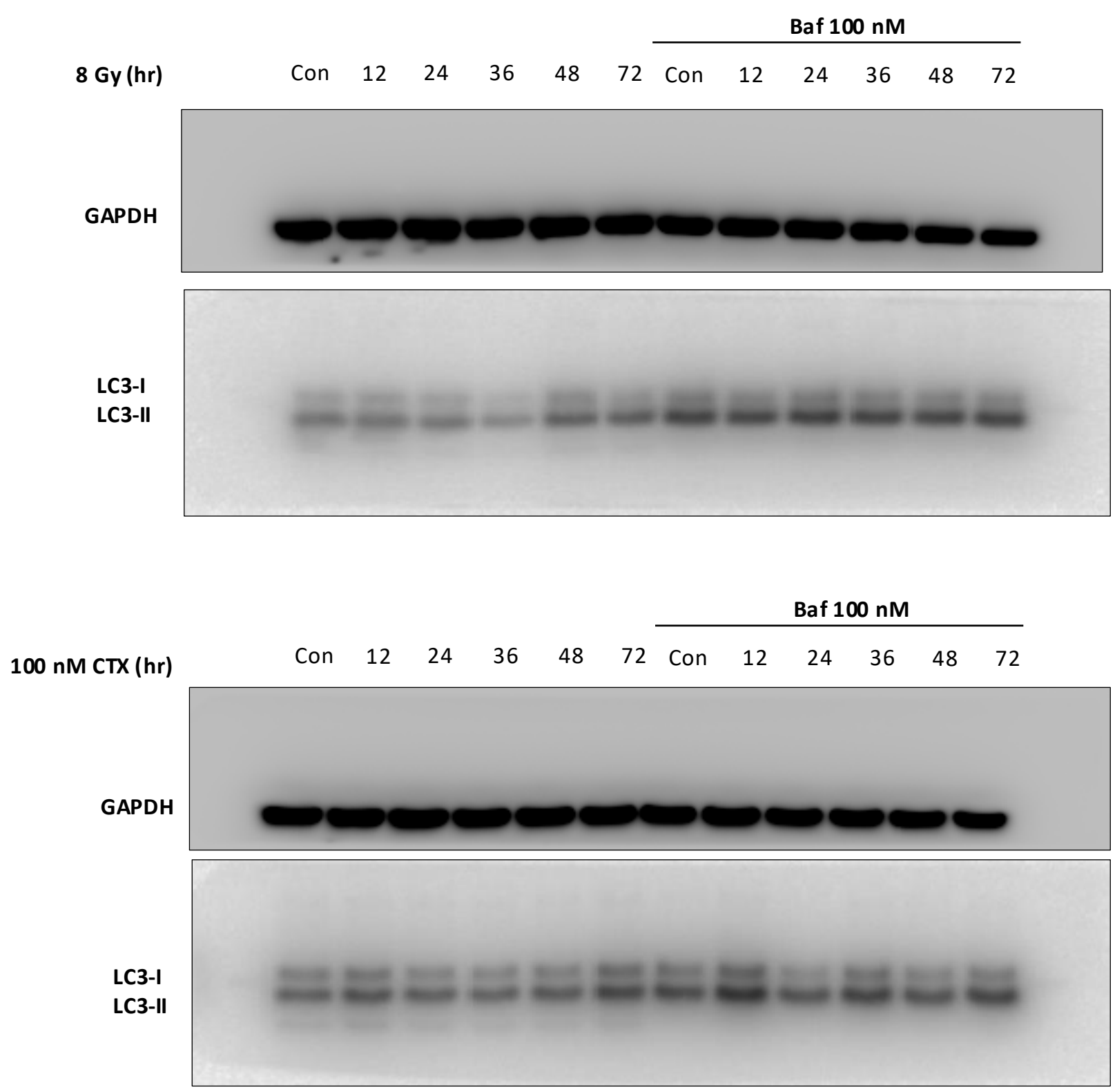

Unprocessed scanned blots matching to the figures

Fig. 1D

UM-SCC47

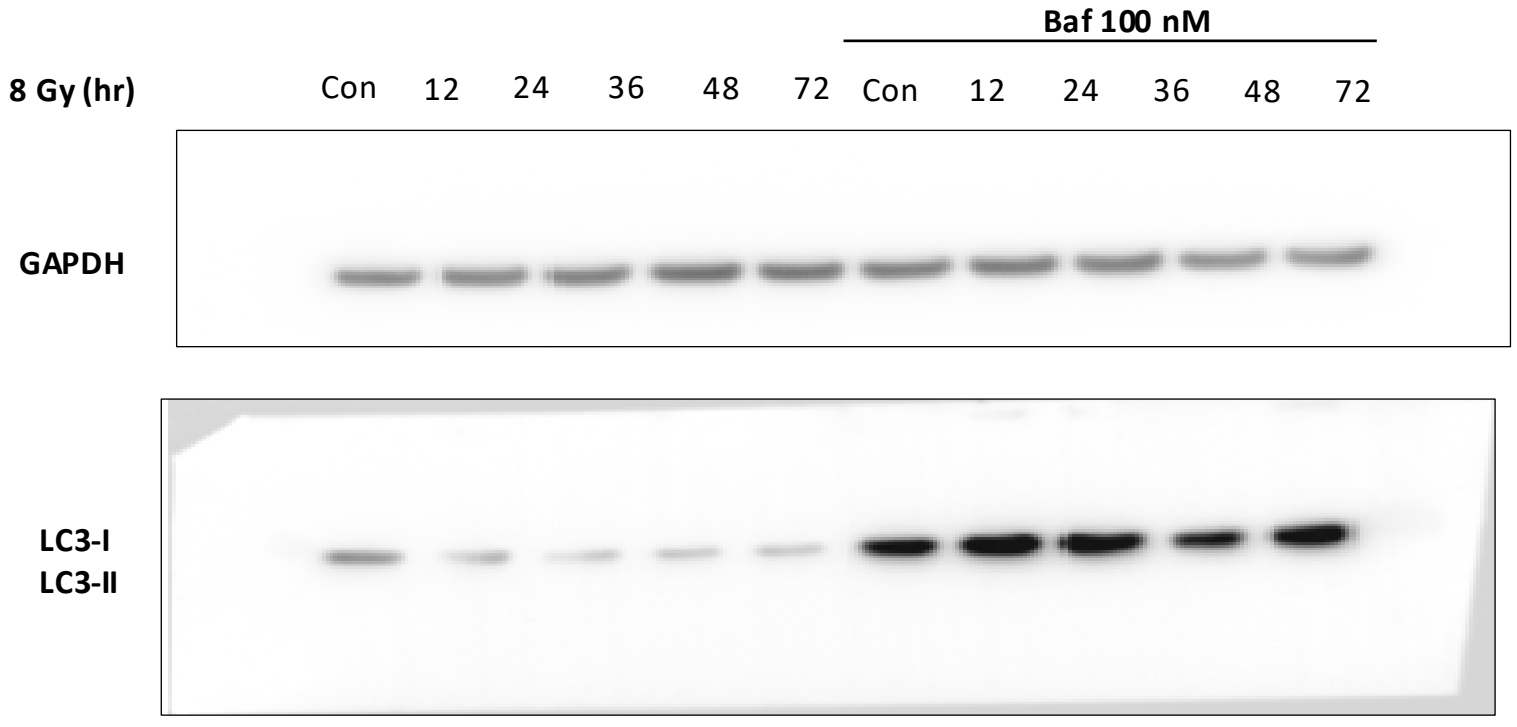

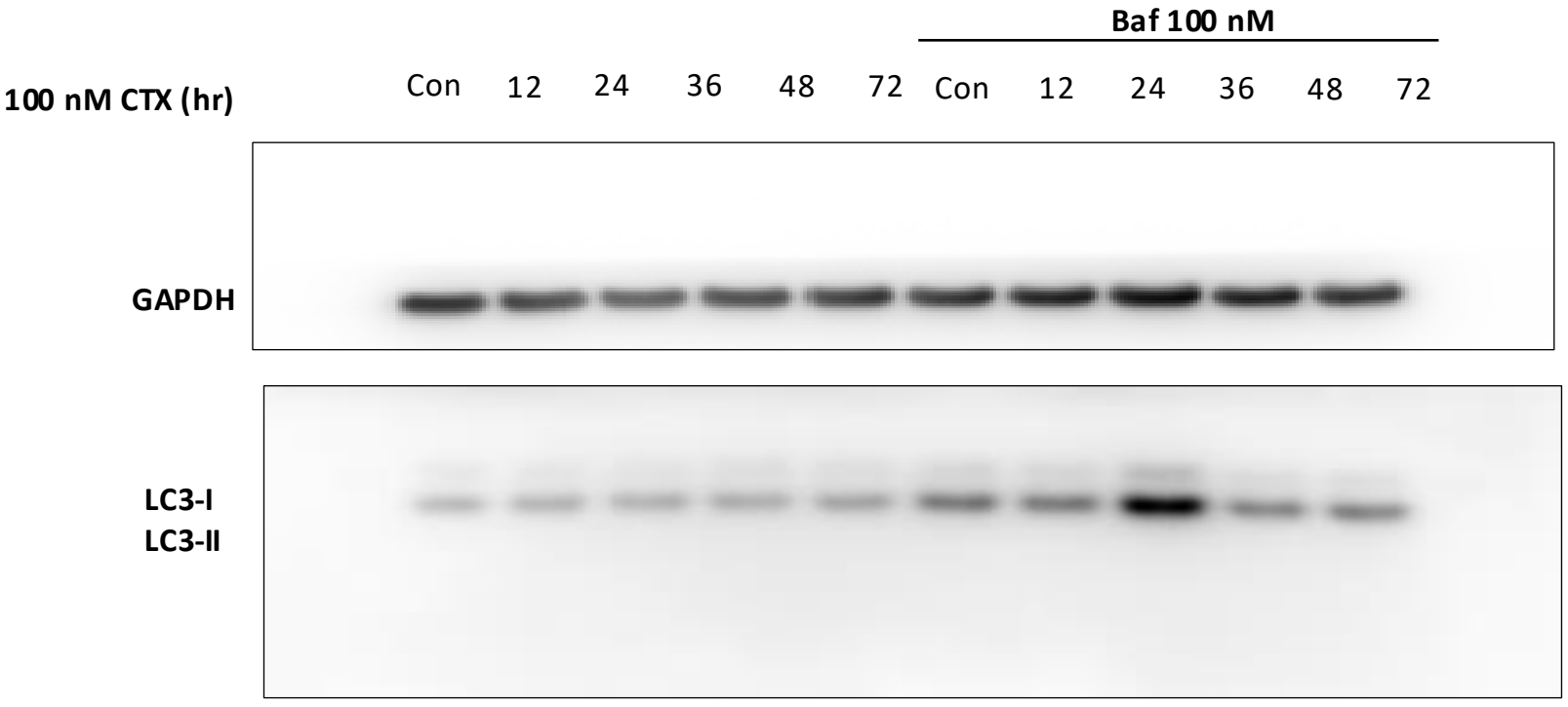

Unprocessed scanned blots matching to the figures

Fig. 3A

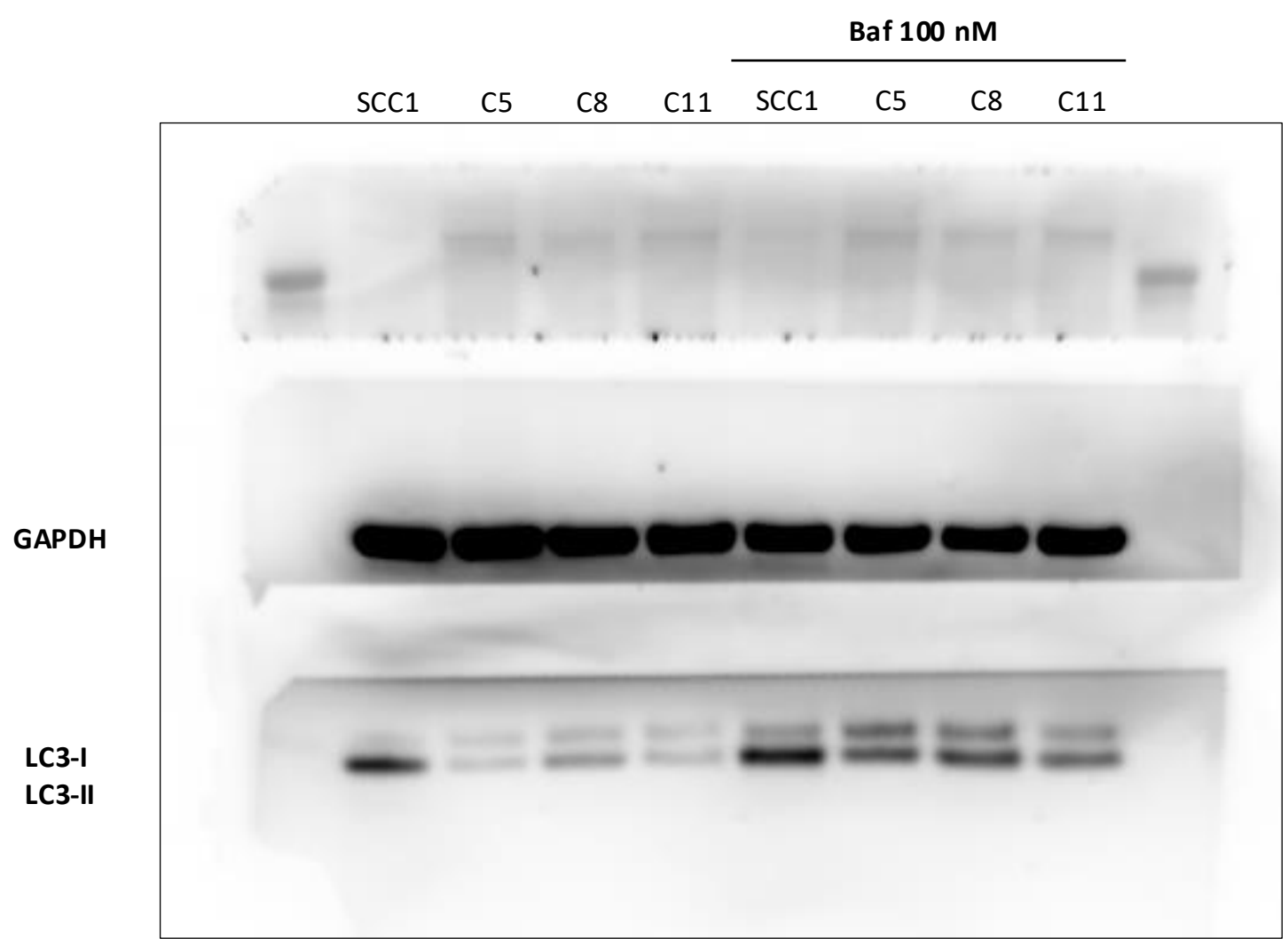

Unprocessed scanned blots matching to the figures

Fig. 3E

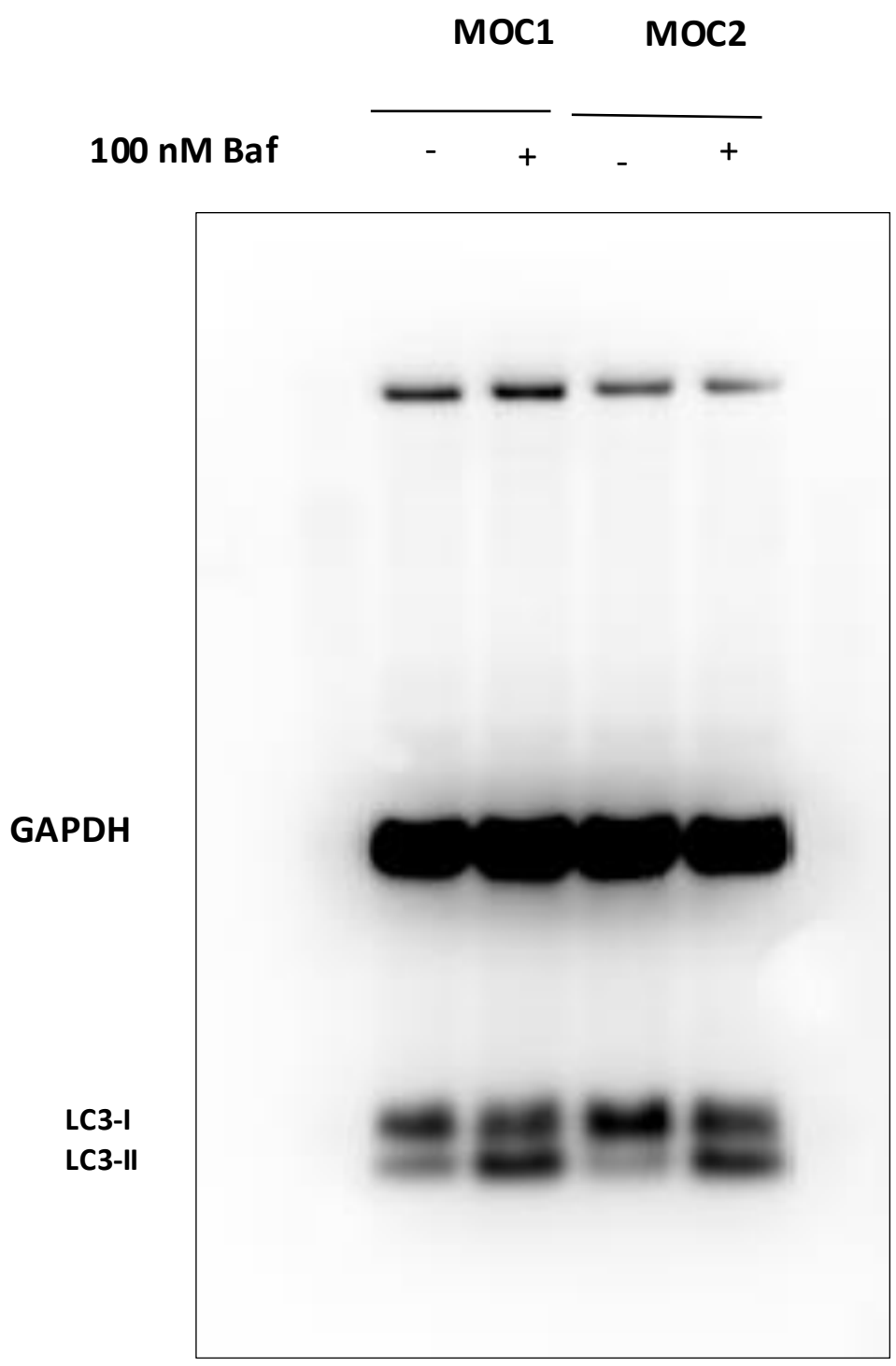

Unprocessed scanned blots matching to the figures

Fig. 5B

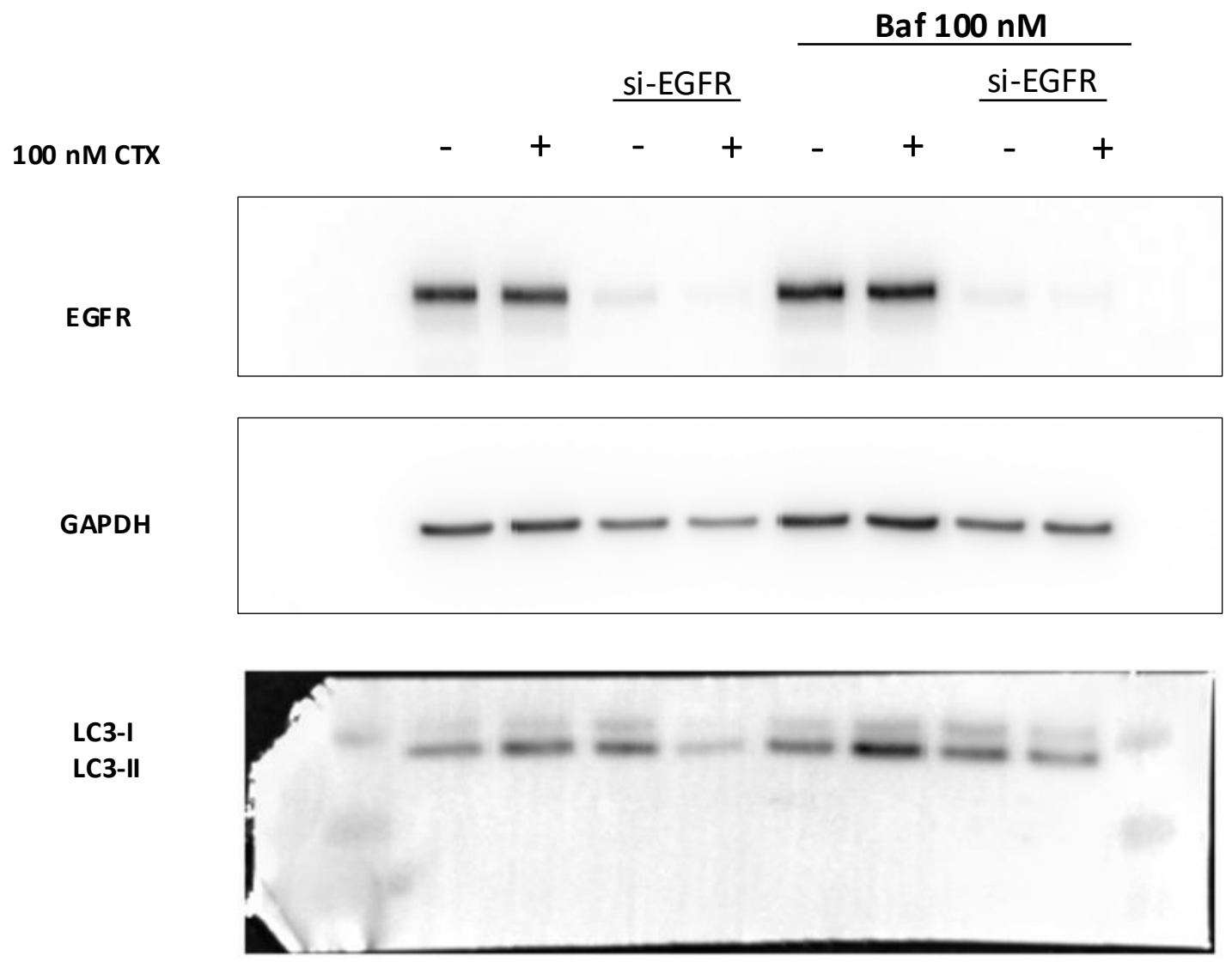

Unprocessed scanned blots matching to the figures

Fig. 6D

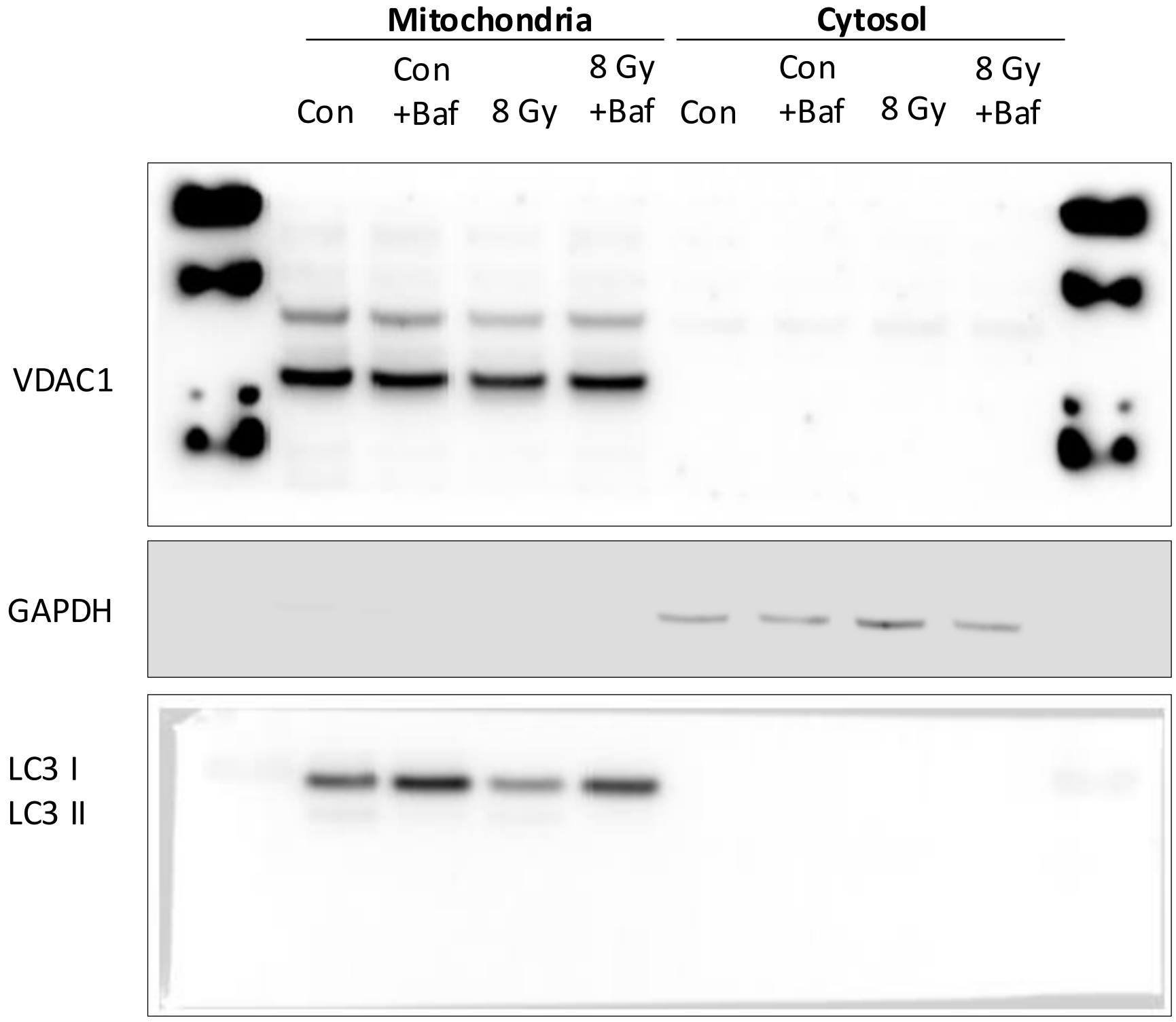

Unprocessed scanned blots matching to the figures

Fig. 6H

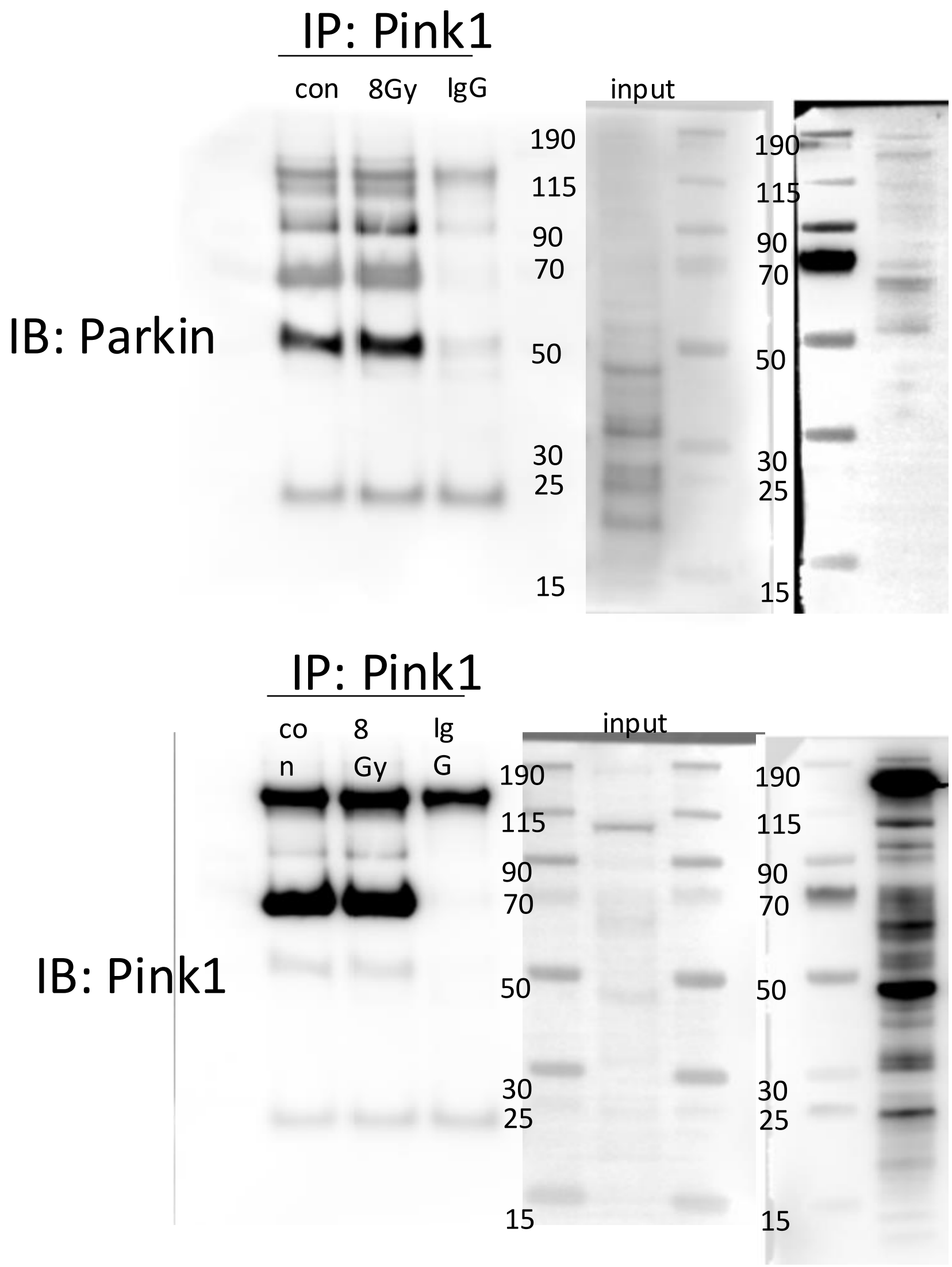

Unprocessed scanned blots matching to the figures

Fig. S2

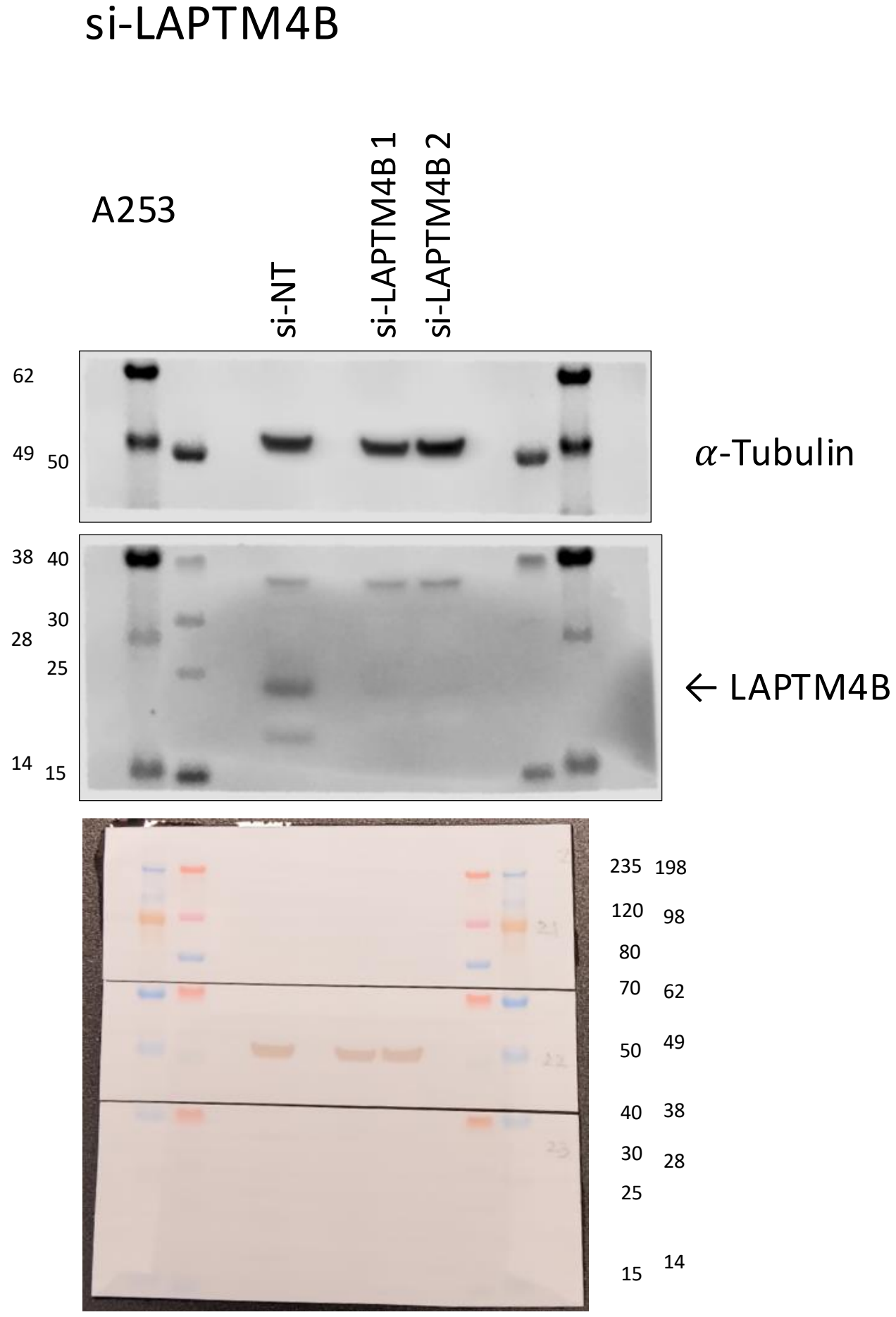

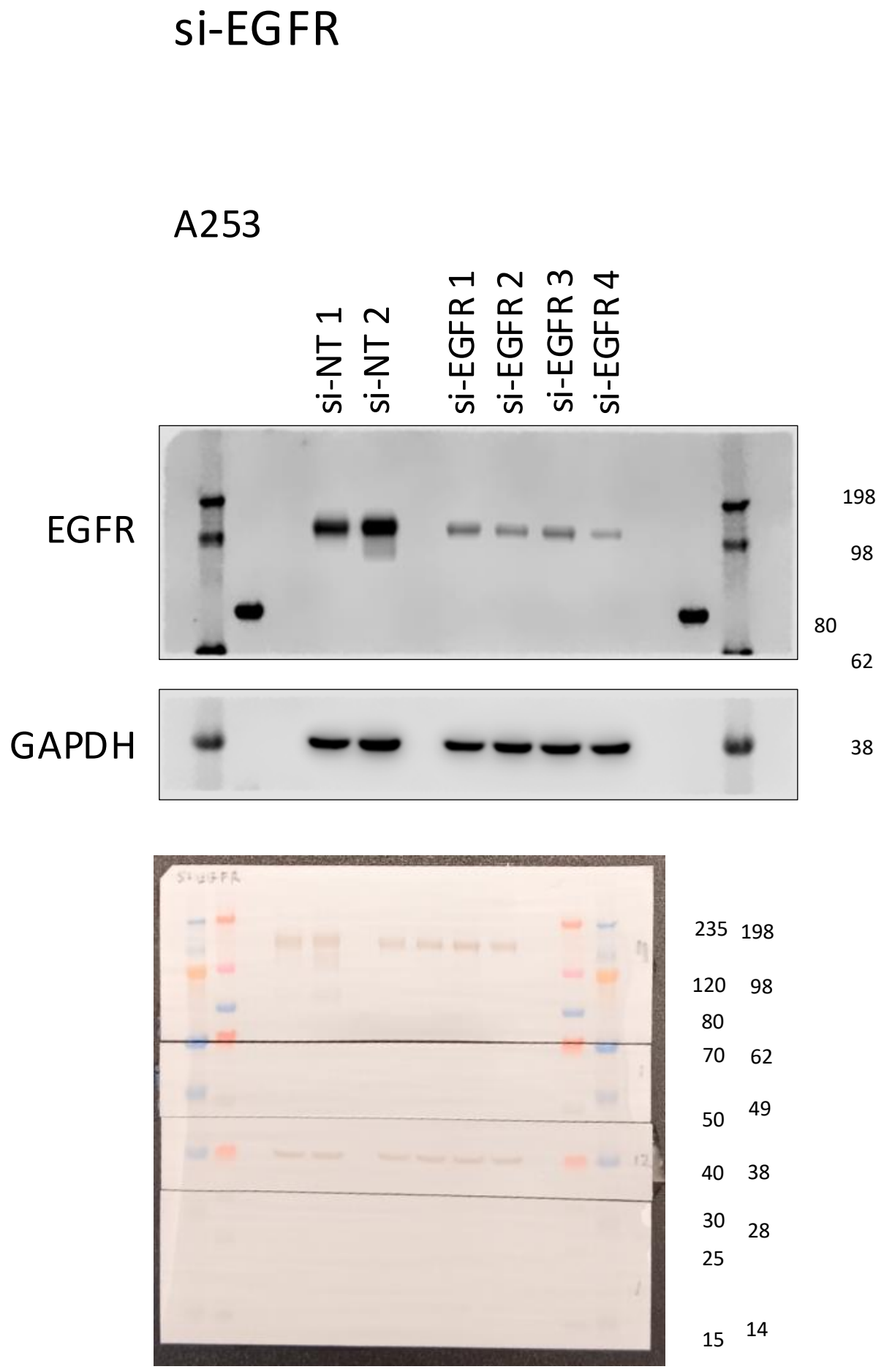

Supplement: Supplementary file 1 [file 41598_2026_53573_MOESM1_ESM.pdf]
